# Subsequent female breast cancer risk associated with anthracycline chemotherapy for childhood cancer

**DOI:** 10.1038/s41591-023-02514-1

**Published:** 2023-09-11

**Authors:** Yuehan Wang, Cécile M. Ronckers, Flora E. van Leeuwen, Chaya S. Moskowitz, Wendy Leisenring, Gregory T. Armstrong, Florent de Vathaire, Melissa M. Hudson, Claudia E. Kuehni, Michael A. Arnold, Charlotte Demoor-Goldschmidt, Daniel M. Green, Tara O. Henderson, Rebecca M. Howell, Matthew J. Ehrhardt, Joseph P. Neglia, Kevin C. Oeffinger, Helena J. H. van der Pal, Leslie L. Robison, Michael Schaapveld, Lucie M. Turcotte, Nicolas Waespe, Leontien C. M. Kremer, Jop C. Teepen, The International Consortium for Pooled Studies on Subsequent Malignancies after Childhood and Adolescent Cancer, Yuehan Wang, Cécile M. Ronckers, Chaya S. Moskowitz, Wendy Leisenring, Gregory T. Armstrong, Melissa M. Hudson, Claudia E. Kuehni, Michael A. Arnold, Charlotte Demoor-Goldschmidt, Daniel M. Green, Tara O. Henderson, Rebecca M. Howell, Matthew J. Ehrhardt, Joseph P. Neglia, Kevin C. Oeffinger, Leslie L. Robison, Michael Schaapveld, Lucie M. Turcotte, Nicolas Waespe, Leontien C. M. Kremer, Jop C. Teepen, Flora E. van Leeuwen, Florent de Vathaire, Helena J. H. van der Pal, Nadia Haddy, Ibrahima Diallo, K. Scott Baker, Amy Berrington de González, Miriam R. Conces, Louis S. Constine, Mike Hawkins, Jacqueline J. Loonen, Marloes Louwerens, Geert O. Janssens, Lene Mellemkjaer, Raoul Reulen, Jeanette F. Winther

**Affiliations:** 1 Princess Máxima Center for Pediatric Oncology, Utrecht, The Netherlands; 2 Department of Health Services Research, Carl von Ossietzky University of Oldenburg, Oldenburg, Germany; 3 Division of Childhood Cancer Epidemiology (EpiKiK), Institute of Medical Biostatistics, Epidemiology and Informatics (IMBEI), University Medical Center of the Johannes Gutenberg University Mainz, Mainz, Germany; 4 Netherlands Cancer Institute, Amsterdam, The Netherlands; 5 Memorial Sloan Kettering Cancer Center, New York City, NY USA; 6 Fred Hutchinson Cancer Center, Seattle, WA USA; 7 St. Jude Children’s Research Hospital, Memphis, TN USA; 8 Radiation Epidemiology Team, INSERM U1018, Gustave Roussy, Université Paris-Saclay, Villejuif, France; 9 Childhood Cancer Research Group, Institute of Social and Preventive Medicine, University of Bern, Bern, Switzerland; 10 Pediatric Hematology and Oncology, University Children’s Hospital Bern, University of Bern, Bern, Switzerland; 11 Department of Pathology and Laboratory Medicine, Children’s Hospital Colorado, Aurora, CO USA; 12 Department of Pathology, University of Colorado Anschutz Medical Campus, Aurora, CO USA; 13 Department of Pediatric Hematology and Oncology, University-Hospital of Angers, Angers, France; 14 Radiotherapy Department, Francois Baclesse Center, Caen, France; 15 University of Chicago Medicine Comer Children’s Hospital, Chicago, IL USA; 16 University of Texas M.D. Anderson Cancer Center, Houston, TX USA; 17 University of Minnesota Masonic Cancer Center, Minneapolis, MN USA; 18 Duke University Medical Center, Durham, NC USA; 19 CANSEARCH research platform in pediatric oncology and hematology of the University of Geneva, Geneva, Switzerland; 20 University Medical Center Utrecht, Wilhelmina Children’s Hospital, Utrecht, The Netherlands; 21 National Cancer Institute, National Institutes of Health, Bethesda, MD USA; 22 Institute of Cancer Research, Sutton, UK; 23 Department of Pathology and Laboratory Medicine, Nationwide Children’s Hospital, Columbus, OH USA; 24 Department of Pathology, The Ohio State University Wexner Medical Center, Columbus, OH USA; 25 University of Rochester Medical Center, Rochester, NY USA; 26 Centre for Childhood Cancer Survivor Studies, University of Birmingham, Birmingham, UK; 27 Radboudumc Center of Expertise for Cancer Survivorship, Department of Hematology, Radboud University Medical Center, Nijmegen, The Netherlands; 28 Department of Internal Medicine, Leiden University Medical Center, Leiden, The Netherlands; 29 Department of Radiation Oncology, University Medical Centre Utrecht, Utrecht, The Netherlands; 30 Danish Cancer Society Research Center, Copenhagen, Denmark; 31 Childhood Cancer Research Group, Danish Cancer Society Research Center, Copenhagen, Denmark; 32 Department of Clinical Medicine, Faculty of Health, Aarhus University and Aarhus University Hospital, Aarhus, Denmark

**Keywords:** Risk factors, Breast cancer, Quality of life, Paediatric cancer, Cancer epidemiology

## Abstract

Anthracycline-based chemotherapy is associated with increased subsequent breast cancer (SBC) risk in female childhood cancer survivors, but the current evidence is insufficient to support early breast cancer screening recommendations for survivors treated with anthracyclines. In this study, we pooled individual patient data of 17,903 survivors from six well-established studies, of whom 782 (4.4%) developed a SBC, and analyzed dose-dependent effects of individual anthracycline agents on developing SBC and interactions with chest radiotherapy. A dose-dependent increased SBC risk was seen for doxorubicin (hazard ratio (HR) per 100 mg m^−^^2^: 1.24, 95% confidence interval (CI): 1.18–1.31), with more than twofold increased risk for survivors treated with ≥200 mg m^−2^ cumulative doxorubicin dose versus no doxorubicin (HR: 2.50 for 200–299 mg m^−^^2^, HR: 2.33 for 300–399 mg m^−^^2^ and HR: 2.78 for ≥400 mg m^−^^2^). For daunorubicin, the associations were not statistically significant. Epirubicin was associated with increased SBC risk (yes/no, HR: 3.25, 95% CI: 1.59–6.63). For patients treated with or without chest irradiation, HRs per 100 mg m^−^^2^ of doxorubicin were 1.11 (95% CI: 1.02–1.21) and 1.26 (95% CI: 1.17–1.36), respectively. Our findings support that early initiation of SBC surveillance may be reasonable for survivors who received ≥200 mg m^−^^2^ cumulative doxorubicin dose and should be considered in SBC surveillance guidelines for survivors and future treatment protocols.

## Abstract (web summary)

In a pooled analysis of six international studies involving about 17,900 female survivors of childhood cancer, the use of doxorubicin was associated with a dose-dependent risk of subsequent breast cancer, irrespective of prior chest radiotherapy exposure.

## Main

Over the past six decades, survival rates for childhood cancer have improved markedly in resource-rich countries due to improvements in treatment and supportive care. Unfortunately, the life expectancy and quality of life of long-term survivors are compromised by long-term adverse effects of treatments such as subsequent neoplasms^1–4^. Breast cancer is one of the most frequent subsequent malignant neoplasms among female childhood cancer survivors^5–7^. Based on strong evidence regarding the effect of chest radiotherapy on subsequent breast cancer (SBC) risk, the International Guideline Harmonization Group (IGHG) recommends initiation of annual breast cancer surveillance for female survivors who received ≥10 Gray (Gy) chest radiotherapy at age 25 years or ≥8 years from radiation^8^, which is earlier than the population screening programs for breast cancer that typically recommend initiation of screening at age 40 years or 50 years^9^.

Over time, childhood cancer treatments have been modified to include decreased radiation doses and volumes and increased use of chemotherapy, especially anthracyclines^10^. Several previous studies have shown that anthracycline exposure is associated with increased SBC risk^7,11–15^, including several Childhood Cancer Survivor Study (CCSS) reports^7,11,14,15^, the St. Jude Lifetime Cohort Study (SJLIFE)^13^ and the Dutch Childhood Cancer Survivor Study LATER (DCCSS-LATER)^12^. These studies investigated the dose-dependent associations between the sum of anthracycline agents dose and SBC risk. Only a few studies evaluated the dose effects of doxorubicin, an individual anthracycline agent, on SBC risk^12,14,16^. However, these studies all used tertiles of cumulative doxorubicin dose, which are derived from cumulative dose distributions that are study dependent. There is currently no data on dose effects with regard to SBC risks for other individual anthracycline agents (for example, daunorubicin). Moreover, there is little information on the joint effects of anthracyclines and chest radiotherapy^14^. The current evidence is insufficient to alter the SBC screening recommendations because there was inconsistent evidence on dose thresholds for determining which survivors are at moderate or high risk. Furthermore, there were no data on possible differences in dose effects with regard to SBC risk for the different individual anthracycline agents owing to the limited number of survivors treated with these specific chemotherapy agents in individual cohort studies.

To address these knowledge gaps, detailed treatment data from a large number of individuals are required. Therefore, we pooled individual patient data from six well-established childhood cancer survivor studies in Europe and North America with the aim of estimating the dose-dependent effects of specific anthracycline agents on developing SBC in female childhood cancer survivors, as well as interactions with chest radiotherapy and age at primary cancer diagnosis.

## Results

**Fig. 1:**
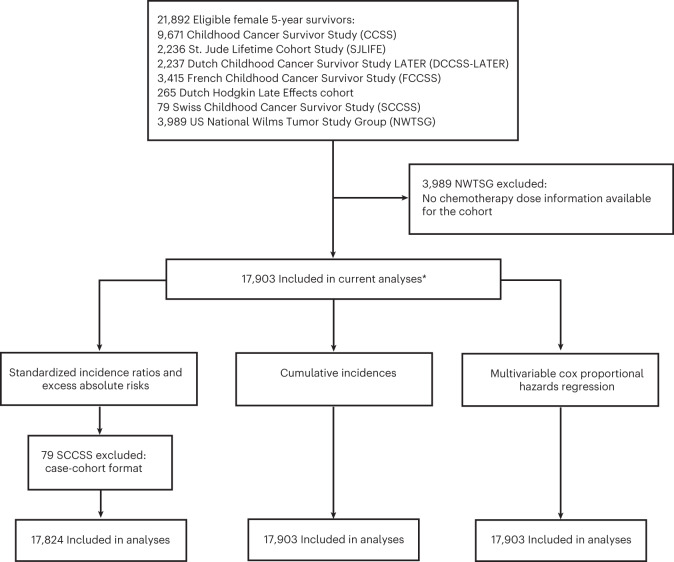
Cohort composition diagram of eligible female 5-year childhood cancer survivors in each analysis. *The number of included survivors in each analysis may vary due to missing values of analysis variables.

In total, our pooled cohort included 17,903 5-year survivors, with data from five cohort studies (CCSS: 9,671 women, SJLIFE: 2,236 women, DCCSS-LATER: 2,237 women, French Childhood Cancer Survivor Study (FCCSS): 3,415 women and Dutch Hodgkin Late Effects cohort: 265 women), and one case–cohort study (Swiss Childhood Cancer Survivor Study (SCCSS): 79 women) in Europe and North America (Fig. 1).

Among the eligible 17,903 5-year survivors, the median age at primary childhood cancer diagnosis was 6.7 years (interquartile range (IQR): 2.8–13.0), with leukemia (25.5%), central nervous system tumor (16.5%) and Hodgkin lymphoma (11.7%) as the most frequent childhood cancer types (Table 1 and Supplementary Tables 1 and 2), with some variations by cohort (Supplementary Table 3). Of all survivors, 5,714 (31.9%) received anthracyclines without chest radiotherapy, 1,962 (11.0%) received chest radiotherapy without anthracyclines, 1,634 (9.1%) received both anthracyclines and chest radiotherapy, 7,096 (39.6%) received neither treatment and for 1,497 (8.4%), it was unclear whether they received anthracycline treatment and/or chest radiotherapy treatment.

**Table 1:** Demographic and treatment characteristics of 17,903 female 5-year childhood cancer survivors (primary cancer diagnosis year 1946–2012) overall and by subsequent breast cancer status

Characteristic	Total (*n* = 17,903)	Subsequent breast cancer^a^ (*n* = 782)	No subsequent breast cancer (*n* = 17,121)
	No. (%)	No. (%)	No. (%)
Primary childhood cancer^b^			
Leukemia	4,574 (25.5)	81 (10.4)	4,493 (26.2)
Non-Hodgkin lymphoma	1,097 (6.1)	37 (4.7)	1,060 (6.2)
Hodgkin lymphoma	2,101 (11.7)	405 (51.8)	1,696 (9.9)
Central nervous system tumor	2,946 (16.5)	14 (1.8)	2,932 (17.1)
Neuroblastoma	1,657 (9.3)	15 (1.9)	1,642 (9.6)
Retinoblastoma	426 (2.4)	2 (0.3)	424 (2.5)
Renal tumor	1,372 (7.7)	45 (5.8)	1,327 (7.8)
Bone tumor	1,459 (8.1)	106 (13.6)	1,353 (7.9)
Soft tissue tumor	1,405 (7.8)	55 (7.0)	1,350 (7.9)
Germ cell tumor	440 (2.5)	9 (1.2)	431 (2.5)
Other malignant epithelial	297 (1.7)	11 (1.4)	286 (1.7)
Other^c^	129 (0.7)	2 (0.2)	127 (0.8)
Cumulative doxorubicin dose (mg m^−2^)			
0	11,170 (62.4)	431 (55.1)	10,739 (62.7)
<100	912 (5.1)	16 (2.0)	896 (5.2)
100–199	1,795 (10.0)	69 (8.8)	1,726 (10.1)
200–299	1,026 (5.7)	67 (8.6)	959 (5.6)
300–399	1,012 (5.7)	64 (8.2)	948 (5.5)
≥400	779 (4.4)	58 (7.4)	721 (4.2)
Unknown^d^	1,209 (6.8)	77 (9.8)	1,132 (6.6)
Cumulative daunorubicin dose (mg m^−^^2^)			
0	14,630 (81.7)	684 (87.5)	13,946 (81.5)
<100	623 (3.5)	7 (0.9)	616 (3.6)
100–199	953 (5.3)	16 (2.0)	937 (5.5)
≥200	645 (3.6)	17 (2.2)	628 (3.7)
Unknown^e^	1,052 (5.9)	58 (7.4)	994 (5.8)
Epirubicin			
No	16,637 (92.9)	717 (91.7)	15,920 (93.0)
Yes	325 (1.8)	9 (1.2)	316 (1.8)
Unknown	941 (5.3)	56 (7.2)	885 (5.2)
Idarubicin			
No	16,843 (94.1)	725 (92.7)	16,118 (94.1)
Yes	107 (0.6)	1 (0.1)	106 (0.6)
Unknown	953 (5.3)	56 (7.2)	897 (5.2)
CED^f^ (mg m^−^^2^)			
0	7,951 (44.4)	301 (38.5)	7,650 (44.7)
<6,000	3,069 (17.1)	94 (12.0)	2,975 (17.4)
6,000–17,999	3,899 (21.8)	192 (24.6)	3,707 (21.7)
≥18,000	1,117 (6.2)	47 (6.0)	1,070 (6.2)
Unknown	1,867 (10.4)	148 (18.9)	1,719 (10.0)
Chest radiotherapy fields and doses^g^			
No chest radiotherapy	13,004 (72.6)	250 (32.0)	12,754 (74.5)
High-dose mantle (≥36 Gy; median: 40 Gy, IQR: 39–44 Gy)^h^	698 (3.9)	238 (30.4)	460 (2.7)
Low-dose mantle (<36 Gy; median: 26 Gy, IQR: 21–30 Gy)^h^	524 (2.9)	93 (11.9)	431 (2.5)
Mediastinal (median: 26 Gy, IQR: 21–36 Gy)^h^	469 (2.6)	33 (4.2)	436 (2.5)
TBI (median: 12 Gy, IQR: 11–13 Gy)^h^	371 (2.1)	22 (2.8)	349 (2.0)
Whole lung (median: 16 Gy, IQR: 12–23 Gy)^h^	184 (1.0)	23 (2.9)	161 (0.9)
Other (median: 28 Gy, IQR: 21–36 Gy)^h^	1,316 (7.4)	63 (8.1)	1,253 (7.3)
Unknown	1,337 (7.5)	60 (7.7)	1,277 (7.5)
Pelvic radiotherapy dose^i^			
No pelvic radiotherapy	13,727 (76.7)	505 (64.6)	13,222 (77.2)
<10 Gy	142 (0.8)	6 (0.8)	136 (0.8)
10–19 Gy	594 (3.3)	24 (3.1)	570 (3.3)
20–29 Gy	719 (4.0)	38 (4.9)	681 (4.0)
30–39 Gy	767 (4.3)	82 (10.5)	685 (4.0)
≥40 Gy	713 (4.0)	72 (9.2)	641 (3.7)
Unknown	1,241 (6.9)	55 (7.0)	1,186 (6.9)
Age at diagnosis of primary cancer (years)			
<5	7,376 (41.2)	66 (8.4)	7,310 (42.7)
5–9	3,788 (21.2)	65 (8.3)	3,723 (21.7)
10–14	3,930 (22.0)	273 (34.9)	3,657 (21.4)
15–21	2,809 (15.7)	378 (48.3)	2,431 (14.2)
Treatment subgroups^j^			
Anthracycline^k^ and chest radiotherapy	1,634 (9.1)	163 (20.8)	1,471 (8.6)
Anthracycline and no chest radiotherapy	5,714 (31.9)	156 (19.9)	5,558 (32.5)
No anthracycline and chest radiotherapy	1,962 (11.0)	294 (37.6)	1,668 (9.7)
No anthracycline and no chest radiotherapy	7,096 (39.6)	83 (10.6)	7,013 (41.0)
Unknown	1,497 (8.4)	86 (11.0)	1,411 (8.2)

^a^Included both invasive and ductal carcinoma in situ breast cancer.

^b^Because of the eligibility criteria of the cohort, the composition of primary cancer diagnosis groups in our pooled data may differ from the composition in underlying populations of childhood cancer survivors.

^c^Included the ICCC-3 classification groups ‘hepatic tumor’ (0 case/61 survivors), ‘other and unspecified’ (1 case/38 survivors), and ‘unclassified’ (1 case/30 survivors).

^d^The unknown category under the variable ‘doxorubicin dose’ included both survivor groups with any doxorubicin (yes/no) unknown (56 cases/941 survivors) and with doxorubicin treatment but dose information unknown (21 cases/268 survivors).

^e^The unknown category under the variable ‘daunorubicin dose’ included both survivor groups with any daunorubicin (yes/no) unknown (56 cases/953 survivors) and with daunorubicin treatment but dose information unknown (2 cases/99 survivors).

^f^CED calculation: CED (mg m^−^^2^) = 1.0 (cumulative cyclophosphamide dose (mg m^−^^2^)) + 0.244 (cumulative ifosfamide dose (mg m^−^^2^)) + 0.857 (cumulative procarbazine dose (mg m^−^^2^)) + 14.286 (cumulative chlorambucil dose (mg m^−^^2^)) + 15.0 (cumulative BCNU (carmustine) dose (mg m^−^^2^)) + 16.0 (cumulative CCNU (lomustine) dose (mg m^−^^2^)) + 40 (cumulative melphalan dose (mg m^−^^2^)) + 50 (cumulative Thio-TEPA (thiotepa) dose (mg m^−^^2^)) + 100 (cumulative nitrogen mustard dose (mg m^−^^2^)) + 8.823 (cumulative busulfan dose (mg m^−^^2^)).

^g^Included radiotherapy fields exposing (parts of) the chest. Radiation dose referred to the cumulative prescribed dose (including boost doses, if applicable), or slight variations, depending on definitions in the underlying cohorts^25^. Chest radiotherapy was categorized as the combination of chest radiation fields with the associated maximum chest radiotherapy dose below or above the median. The variable was classified as follows: high-dose mantle (median: 40 Gy, IQR: 39–44 Gy), low-dose mantle (median: 26 Gy, IQR: 21–30 Gy), mediastinal (median: 26 Gy, IQR: 21–36 Gy), TBI (median: 12 Gy, IQR: 11–13 Gy), whole lung (median: 16 Gy, IQR: 12–23 Gy), other (median: 28 Gy, IQR: 21–36 Gy) and unknown.

^h^Dose represents the maximum cumulative prescribed chest dose (including boost doses, if applicable) of survivors classified in this group. This could include doses to chest field other than this category.

^i^Included radiotherapy fields exposing (parts of) the pelvis (including TBI). Radiation dose referred to the cumulative prescribed dose (including boost doses, if applicable), or slight variations, depending on definitions in the underlying cohorts^25^. The unknown category under the variable ‘pelvic radiotherapy dose’ included both survivor groups with any pelvic radiotherapy (yes/no) unknown (54 cases/1,212 survivors) and with pelvic radiotherapy treatment but dose information unknown (1 case/29 survivors).

^j^Treatment subgroup variable set to unknown if either of the treatment categories was unknown.

^k^Anthracyclines included doxorubicin, daunorubicin, epirubicin and idarubicin.

Among survivors with a cumulative doxorubicin dose of ≥200 mg m^−2^, the most common childhood cancer types were bone tumors, Hodgkin lymphoma and soft tissue sarcomas (Extended Data Table 1). The highest percentage of survivors who received a cumulative doxorubicin dose of ≥200 mg m^−2^ was observed in the Dutch Hodgkin Late Effects cohort (20.8%), followed by the CCSS (16.9%) and the FCCSS (16.0%). Although the percentage of survivors who received any doxorubicin treatment was higher in the diagnosis period ≥1990 (39.3%) compared to the diagnosis periods 1980–1989 (33.0%) and <1980 (20.2%), the percentage of survivors who received ≥200 mg m^−2^ cumulative doxorubicin dose was fairly similar across diagnosis period ≥1990 (14.3%), diagnosis period 1980–1989 (18.6%) and diagnosis period <1980 (14.2%). The median follow-up time after primary cancer diagnosis was 24.9 years (IQR: 19.1–33.2). In total, 782 survivors developed the first SBC at a median age of 39.7 years (IQR: 34.3–44.9), including 616 invasive breast cancer and 166 ductal carcinoma in situ (DCIS) cases. The median attained age at the end of follow-up was 33.7 years (IQR: 25.9–41.6) and 29.6% of survivors attained an age of 40 years or more.

### Comparison with the general population

In Extended Data Table 2, breast cancer risk by doxorubicin and chest radiotherapy treatment is provided. Compared with the general female population, the risk of invasive breast cancer was most elevated in the survivor group that received a cumulative doxorubicin dose ≥200 mg m^−^^2^ and chest radiotherapy (standardized incidence ratio (SIR): 17.5, 95% confidence interval (CI): 13.3–22.6; median attained age, 36.1 years), followed by the cumulative doxorubicin dose <200 mg m^−^^2^ and chest radiotherapy group (SIR: 13.9, 95%: CI: 9.7–19.2; median attained age, 33.8 years), then by the chest radiotherapy-only with no doxorubicin group (SIR: 10.7, 95%: CI 9.4–12.1; median attained age, 38.4 years), then by the cumulative doxorubicin dose ≥200 mg m^−^^2^ with no chest radiotherapy group (SIR: 5.6, 95% CI: 4.5–6.9; median attained age, 36.5 years), then by the cumulative doxorubicin dose <200 mg m^−^^2^ with no chest radiotherapy group (SIR: 3.2, 95% CI: 1.9–5.1; median attained age, 28.9 years) and finally by the group receiving neither doxorubicin nor chest radiotherapy (SIR: 1.7, 95% CI: 1.4–2.1; median attained age, 32.8 years). The highest excess absolute risk (EAR) was observed in the cumulative doxorubicin dose ≥200 mg m^−^^2^ and chest radiotherapy group with 5.0 excess cases per 1,000 person-years.

### Risk factors for SBC

In multivariable Cox regression analyses, cumulative doxorubicin dose was associated with an increased risk of SBC, with a hazard ratio (HR) of 1.76 (95% CI: 0.88–3.51) for <100 mg m^−^^2^, HR of 1.77 (95% CI: 1.30–2.42) for 100–199 mg m^−^^2^, HR of 2.50 (95% CI: 1.85–3.40) for 200–299 mg m^−^^2^, HR of 2.33 (95% CI: 1.68–3.23) for 300–399 mg m^−^^2^ and HR of 2.78 (95% CI: 1.99–3.88) for ≥400 mg m^−^^2^ cumulative doxorubicin dose category compared to the no doxorubicin treatment (Table 2 Model I; survivor characteristics by cumulative doxorubicin dose categories are shown in Extended Data Table 1). Compared to those not treated with daunorubicin, HRs were close to one for those with cumulative doses of daunorubicin <200 mg m^−2^ (HR: 0.98, 95% CI: 0.46–2.09 for <100 mg m^−2^ and HR: 0.98, 95% CI: 0.55–1.75 for 100–199 mg m^−2^), and the highest cumulative dose group, ≥200 mg m^−2^, conferred a nonstatistically significant association (HR: 1.22, 95% CI: 0.69–2.17). When the continuous cumulative doxorubicin and daunorubicin dose information was included in the model, the risk of developing SBC in survivors treated with doxorubicin increased 1.24-fold (HR per 100 mg m^−2^ 1.24, 95% CI: 1.18–1.31) for every 100 mg m^−2^ increase in cumulative doxorubicin dose after adjustments (Table 2, model II). Cumulative daunorubicin dose and risk of SBC were not statistically significant (HR per 100 mg m^−2^ 1.10, 95% CI: 0.95–1.29). Epirubicin treatment was associated with an increased SBC risk (yes versus no, HR: 3.25, 95% CI: 1.59–6.63).

**Table 2:** Multivariable Cox proportional hazard regression analyses for subsequent breast cancer in female 5-year childhood cancer survivors (primary cancer diagnosis year 1946–2012)

Characteristic	Total (*n*)	%	No. of SBC (*n*)^c^	%	Model I^a^		Model II^b^	
					HR	95% CI	HR	95% CI
Cumulative doxorubicin dose (mg m^−^²)								
0	11,170	62.4	431	55.1	1.0	Ref.	–	
<100	912	5.1	16	2.0	1.76	0.88–3.51		
100–199	1,795	10.0	69	8.8	1.77	1.30–2.42		
200–299	1,026	5.7	67	8.6	2.50	1.85–3.40		
300–399	1,012	5.7	64	8.2	2.33	1.68–3.23		
≥400	779	4.4	58	7.4	2.78	1.99–3.88		
Unknown	1,209	6.8	77	9.8	–	–		
Continuous variable: cumulative doxorubicin dose (per 100 mg m^−^²)	–	–	–	–	–	–	1.24	1.18–1.31
Cumulative daunorubicin dose (mg m^−^²)								
0	14,630	81.7	684	87.5	1.0	Ref.	–	
<100	623	3.5	7	0.9	0.98	0.46–2.09		
100–199	953	5.3	16	2.0	0.98	0.55–1.75		
≥200	645	3.6	17	2.2	1.22	0.69–2.17		
Unknown	1,052	5.9	58	7.4	–	–		
Continuous variable: cumulative daunorubicin dose (per 100 mg m^−^²)	–	–	–	–	–	–	1.10	0.95–1.29
Epirubicin								
No	16,637	92.9	717	91.7	1.0	Ref.	1.0	Ref.
Yes	325	1.8	9	1.2	3.40	1.66–6.98	3.25	1.59–6.63
Unknown	941	5.3	56	7.2	–	–	–	–
Chest radiotherapy field and dose								
No chest radiotherapy	13,004	72.6	250	32.0	1.0	Ref.	1.0	Ref.
High-dose mantle (≥36 Gy; median: 40 Gy, IQR: 39–44 Gy)	698	3.9	238	30.4	8.99	7.00–11.53	9.12	7.09–11.75
Low-dose mantle (<36 Gy; median: 26 Gy, IQR: 21–30 Gy)	524	2.9	93	11.9	4.72	3.48–6.41	5.23	3.86–7.09
Mediastinal (median: 26 Gy, IQR: 21–36 Gy)	469	2.6	33	4.2	1.65	1.02–2.67	1.71	1.06–2.78
TBI (median: 12 Gy, IQR: 11–13 Gy)	371	2.1	22	2.8	7.05	4.11–12.10	7.18	4.18–12.33
Whole lung (median: 16 Gy, IQR: 12–23 Gy)	184	1.0	23	2.9	7.58	4.68–12.27	8.00	4.94–12.95
Other (median: 28 Gy, IQR: 21–36 Gy)	1,316	7.4	63	8.1	2.61	1.87–3.64	2.68	1.91–3.75
Unknown	1,337	7.5	60	7.7	–	–	–	–
Pelvic radiotherapy ≥5 Gy								
No	13,751	76.8	505	64.6	1.0	Ref.	1.0	Ref.
Yes	2,911	16.3	222	28.4	0.95	0.78–1.17	0.94	0.77–1.16
Unknown	1,241	6.9	55	7.0	–	–	–	–
Age at primary childhood cancer diagnosis (year)								
<5	7,376	41.2	66	8.4	1.0	Ref.	1.0	Ref.
5–9	3,788	21.2	65	8.3	1.13	0.76–1.69	1.12	0.75–1.67
10–14	3,930	22.0	273	34.9	2.03	1.48–2.79	2.04	1.49–2.81
15–21	2,809	15.7	378	48.3	1.83	1.31–2.55	1.84	1.32–2.57
CED^d^ (mg m^−2^)								
None	7,951	44.4	301	38.5	1.0	Ref.	1.0	Ref.
<6,000	3,069	17.1	94	12.0	0.87	0.67–1.14	0.95	0.73–1.25
6,000–17,999	3,899	21.8	192	24.6	1.02	0.82–1.27	1.07	0.86–1.32
≥18,000	1,117	6.2	47	6.0	1.20	0.83–1.74	1.23	0.85–1.77
Unknown	1,867	10.4	148	18.9	–	–	–	–

^a^Model I included categorical variables of cumulative doxorubicin and daunorubicin dose by steps of 100 mg m^−2^.

^b^Model II included continuous variables of cumulative doxorubicin and daunorubicin dose per 100 mg m^−2^.

^c^One survivor had SBC before 5 years after primary cancer.

^d^CED calculation: CED (mg m^−^^2^) = 1.0 (cumulative cyclophosphamide dose (mg m^−^^2^)) + 0.244 (cumulative ifosfamide dose (mg m^−^^2^)) + 0.857 (cumulative procarbazine dose (mg m^−^^2^)) + 14.286 (cumulative chlorambucil dose (mg m^−^^2^)) + 15.0 (cumulative BCNU (carmustine) dose (mg m^−^^2^)) + 16.0 (cumulative CCNU (lomustine) dose (mg m^−^^2^)) + 40 (cumulative melphalan dose (mg m^−^^2^)) + 50 (cumulative Thio-TEPA (thiotepa) dose (mg m^−^^2^)) + 100 (cumulative nitrogen mustard dose (mg m^−^^2^)) + 8.823 (cumulative busulfan dose (mg m^−^^2^)).

Additionally, all chest radiotherapy field and dose categories were significantly associated with increased SBC risk, with the highest HRs for those treated with high-dose mantle field (HR: 8.99, 95% CI: 7.00–11.53), followed by whole lung irradiation (HR: 7.58, 95% CI: 4.68–12.27), and total body irradiation (TBI; HR: 7.05, 95% CI: 4.11–12.10; Table 2, model I). Survivors with a primary cancer diagnosis at ages 10–14 or 15–21 years had an elevated risk of SBC with HRs of 2.03 (95% CI: 1.48–2.79) for 10–14 years and 1.83 (95% CI: 1.31–2.55) for 15–21 years compared with the survivors who were diagnosed at ages 0–4. We did not observe significant effects of pelvic radiotherapy or alkylating agents (cyclophosphamide equivalent dose (CED)) on SBC risk. HR per 100 mg m^−2^ of cumulative doxorubicin dose was 1.11 (95% CI: 1.02–1.21) for survivors who received chest radiotherapy and 1.26 (95% CI: 1.17–1.36) for survivors who did not receive chest radiotherapy (Table 3).

**Table 3:** Multivariable Cox proportional hazard regression analyses for subsequent breast cancer by chest radiotherapy status among female 5-year childhood cancer survivors (primary cancer diagnosis year 1946–2012)

Models without interaction				
Characteristic	With chest radiotherapy^a^		Without chest radiotherapy^a^	
	HR	95% CI	HR	95% CI
Continuous variable: cumulative doxorubicin dose (per 100 mg m^−^²)	1.11	1.02–1.21	1.26	1.17–1.36
Continuous variable: cumulative daunorubicin dose (per 100 mg m^−^²)	0.95	0.74–1.21	1.12	0.93–1.36
Epirubicin				
No	1.0	Ref.	1.0	Ref.
Yes	2.28	1.00–5.21	2.13	0.49–9.17
**Models with a multiplicative interaction**				
–	**Interaction: cumulative doxorubicin dose** **×** **chest radiotherapy status (yes/no)**		**Interaction: cumulative daunorubicin dose** **×** **chest radiotherapy status (yes/no)**	
	HR	95% CI	HR	95% CI
Continuous variable: Cumulative doxorubicin dose (per 100 mg m^−^²)	1.28	1.21–1.37	1.21	1.15–1.28
Continuous variable: cumulative daunorubicin dose (per 100 mg m^−^²)	1.04	0.89–1.23	1.15	0.96–1.37
Epirubicin				
No	1.0	Ref.	1.0	Ref.
Yes	2.54	1.22–5.30	2.47	1.17–5.23
Interaction: cumulative doxorubicin dose (per 100 mg m^−^^2^) × chest radiotherapy status (yes/no)	0.86	0.78–0.96	–	–
Interaction: cumulative daunorubicin dose (per 100 mg m^−2^) × chest radiotherapy status (yes/no)	–	–	0.77	0.57–1.05

^a^Models were further adjusted for pelvic radiotherapy ≥5 Gy (yes/no), age at primary childhood cancer diagnosis (categorical variable), and cyclophosphamide equivalent dose (categorical variable).

Joint effects of continuous cumulative doxorubicin dose and chest radiation (yes versus no) were submultiplicative (HR_multiplicative interaction_: 0.86, 95% CI: 0.78–0.96, *P*_multiplicative interaction_ = 0.006) and compatible with additive effects (*P*_additive interaction_ = 0.99; Extended Data Table 3). The effect of cumulative doxorubicin dose on SBC risk was significantly less strong among those with high-dose mantle field (HR_multiplicative interaction_: 0.84, 95% CI: 0.71–0.98, *P*_multiplicative interaction_ = 0.03) and mediastinal field irradiation (HR_multiplicative interaction_: 0.65, 95% CI: 0.45–0.96, *P*_multiplicative interaction_ = 0.03), compared to those treated without chest radiotherapy. On an additive scale, the joint effects of cumulative doxorubicin dose and chest radiotherapy fields were equal to the sum of these two individual effects (all *P*_additive interaction_ > 0.05). Joint effects of daunorubicin and chest radiation were on a multiplicative scale (*P*_multiplicative interaction_ = 0.10) and significantly less than additive (no. of additional cases per 10,000 person-years: −9.67, *P*_additive interaction_ = 0.002).

Age at childhood cancer diagnosis did not significantly modify the effects of cumulative doxorubicin and daunorubicin dose on SBC risk on a multiplicative scale (*P*_multiplicative interaction_ = 0.09 and *P*_multiplicative interaction_ = 0.30, respectively). However, on an additive scale, the joint effects of cumulative doxorubicin dose and age at childhood cancer diagnosis (5–9; 10–14; 15–21 versus 0–4 years) were all significantly greater than the sum of the individual effects (all *P*_additive interaction_ < 0.05). Such an effect was not found for cumulative daunorubicin dose.

To rule out potential effects of other treatments that have been associated with SBC, such as chest radiotherapy and alkylating agents, we performed separate analyses in survivors who received neither chest radiotherapy nor alkylating agents; the effects of high cumulative doxorubicin dose on SBC risk remained statistically significant, with HR of 2.67 (95% CI: 1.08–6.59) for 300–399 mg m^−2^ and HR of 3.58 (95% CI: 1.66–7.71) for ≥400 mg m^−2^ cumulative doxorubicin dose category (Table 4).

**Table 4:** Multivariable Cox proportional hazard regression analyses for subsequent breast cancer in female 5-year childhood cancer survivors whose treatment history did not include chest radiotherapy nor alkylating agent chemotherapy (primary cancer diagnosis year 1946–2012)^a^

	Total (*n*)	%	No. of SBC (*n*)	%	HR^b^	95% CI
Cumulative doxorubicin dose (mg m^−^²)						
0	5,880	90.5	61	71.8	1.0	Ref.
<300^c^	305	4.7	7	8.2	2.35	0.96–5.71
300–399	131	2.0	5	5.9	2.67	1.08–6.59
≥400	136	2.1	8	9.4	3.58	1.66–7.71
Unknown	47	0.7	4	4.7	–	–
Daunorubicin						
No	6,020	92.6	81	95.3	1.0	Ref.
Yes	479	7.4	4	4.7	1.43	0.48–4.24
Epirubicin						
No	6,362	97.9	84	98.8	1.0	Ref.
Yes	137	2.1	1	1.2	3.46	0.41–29.10

^a^In total, 6,499 female 5-year survivors did not receive chest radiotherapy nor alkylating agent chemotherapy, among whom 85 developed SBC during follow-up.

^b^Models were further adjusted for pelvic radiotherapy ≥5 Gy (yes/no) and age at primary childhood cancer diagnosis (categorical variable).

^c^The cumulative doxorubicin dose (mg m^−2^) categories <100 (0 case/46 survivors), 100–199 (1 case/129 survivors) and 200–299 (6 cases/130 survivors) were collapsed due to low numbers of SBC cases.

### Cumulative incidences

**Fig. 2:**
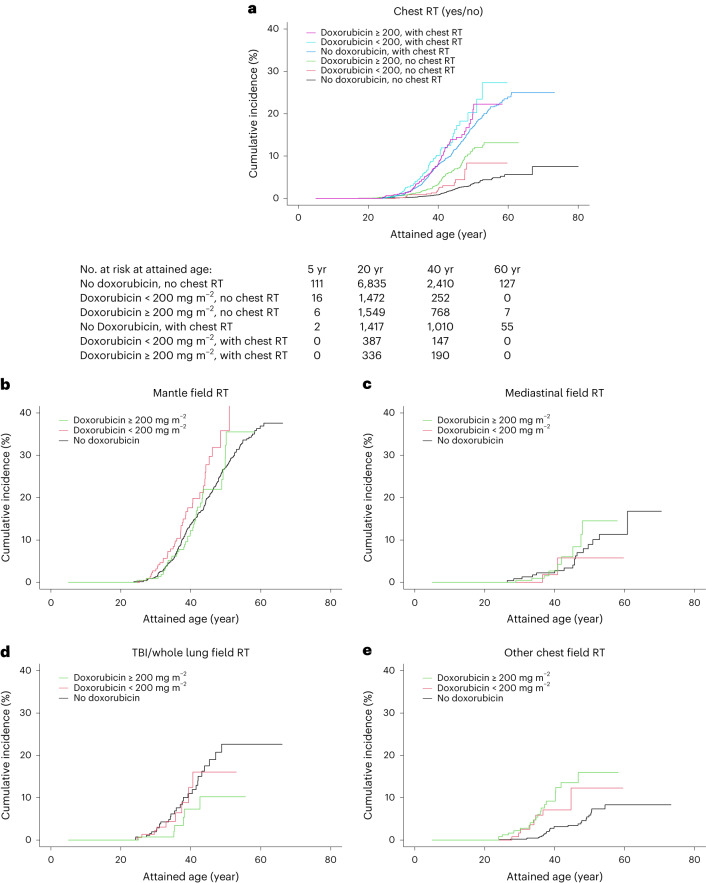
Cumulative incidence of subsequent breast cancer in female 5-year childhood cancer survivors by cumulative doxorubicin dose, stratified by chest radiotherapy status (primary cancer diagnosis year 1946–2012). Stratification by **a**, chest radiotherapy status; **b**, Mantle field; **c**, Mediastinal field; **d**, TBI/whole lung field; **e**, Other chest field. The SCCSS was excluded from cumulative incidence analyses due to the case–cohort design. The cumulative incidence figures represent univariable comparisons. Multivariable Cox regression results for the cumulative doxorubicin dose categories presented in this figure are shown in Extended Data Table 4. No., number; RT, radiotherapy.

For survivors who did not receive chest radiotherapy, cumulative incidences at the age of 40 years were 0.8% for no doxorubicin treatment group, 1.9% for <200 mg m^−2^ cumulative doxorubicin dose group and 3.4% for ≥200 mg m^−2^ cumulative doxorubicin dose group; for survivors who received chest radiotherapy, corresponding cumulative incidences at age 40 for the three cumulative dose groups were 7.9% for no doxorubicin treatment group, 10.1% for <200 mg m^−2^ cumulative doxorubicin dose group and 8.1% for ≥200 mg m^−2^ cumulative doxorubicin dose group (Fig. 2a), with some variation by chest radiation field (Fig. 2b–e). In Extended Data Table 4, multivariable Cox regression results for these cumulative doxorubicin dose categories are presented.

### Sensitivity analyses

Sensitivity analyses including (a) only invasive breast cancer as an outcome, (b) censoring at the time of first non-SBC subsequent malignant tumor, (c) excluding females treated before 1970, (d) excluding patients with Hodgkin lymphoma (all in Extended Data Table 5) and (e) excluding each cohort on a one-by-one basis (Supplementary Table 4) yielded similar results. The results of the models conducted in each cohort are shown in Supplementary Table 5.

## Discussion

Previous studies based on single cohorts reported that anthracycline exposure may increase the risk of SBC, but had relatively smaller sample sizes and case numbers^7,11–15^. We are able to estimate precise dose thresholds for doxorubicin and identify the effects of other types of anthracyclines on SBC risk in a pooled cohort analysis of large numbers of childhood cancer survivors. These pooled analyses demonstrate a relationship between increasing cumulative doxorubicin dose and SBC risk, as well as an association between epirubicin exposure (yes versus no) and an increased SBC risk. We observed that treatment with doxorubicin increases SBC risk both in survivors who received chest radiotherapy and in survivors treated without chest radiotherapy. Furthermore, the joint effects between doxorubicin and chest radiotherapy appear to be additive. In addition, our results did not show a statistically significant association between daunorubicin and increased SBC risk.

The mechanisms underpinning anthracycline-related SBC risk have not been elucidated. Known mutagenic properties of anthracycline agents that might contribute to SBC risk include topoisomerase II inhibition, DNA intercalation, oxidative stress and chromatin damage^17^. In regard to our identification of differential risk between different anthracycline agents, potential differences in the mechanisms of developing subsequent neoplasms are unclear. Animal studies indicate that both doxorubicin and daunorubicin can induce mammary tumors^18,19^. The antineoplastic properties of doxorubicin and daunorubicin have both been assumed to result from DNA damage and chromatin damage^17^, and based on limited studies, the anticancer efficacies are thought to be similar^20,21^. Evidence from murine models and human cells suggests that chemically separating those activities by reducing the DNA damage effect while retaining chromatin damage could detoxify the anthracycline variants while maintaining anticancer efficacy^22^. A possible factor that might underlie the differences in dose effects observed between doxorubicin and daunorubicin is the lower number of individuals and SBC cases among those exposed to daunorubicin, which might have limited power to detect a substantial dose–response relationship. For epirubicin (nine SBC cases exposed), we identified an association with SBC increased risk, but for idarubicin (one SBC case exposed), the number of cases was too low. Future experimental and animal studies that elucidate mechanisms underlying breast carcinogenicity among the various anthracycline agents are warranted.

Childhood cancer treatments often feature multimodality regimens^23^, which challenge the elucidation of joint and independent effects of different treatments. Our study provides evidence of the joint effects of chest radiotherapy and individual anthracycline agents. Our findings indicate that the joint effects of doxorubicin and chest radiation are submultiplicative and compatible with additive effects, which implies that the combined effects of doxorubicin and chest radiation are not equal to the product of their individual effects, but to the sum of their individual effects. A previous CCSS case–control study showed that the joint effects of radiotherapy dose to the breast and anthracycline exposure (yes/no) were more than additive^14^. However, they did not investigate the interaction between individual anthracycline agents and chest radiotherapy, and further comparison between the studies is difficult because the CCSS study used a case–control design with estimated radiation dose to breast cancer location.

We did not observe a statistically significant reduction of SBC risk among survivors with radiotherapy delivered to the pelvic region (≥ 5 Gy versus no pelvic radiotherapy or <5 Gy, as an indicator of ovarian dose) in our entire cohort (Table 2), which aligns with a SJLIFE study (pelvic radiotherapy yes versus no) that was also included in our pooled cohort^13^. However, when we restricted our analyses to survivors who received chest radiotherapy (Extended Data Table 6), we found a decreased SBC risk for pelvic radiotherapy, consistent with previous reports that showed reduced SBC risk associated with absorbed ovarian radiation dose ≥5 Gy in survivors treated with chest radiation, likely due to suppression of hormonal stimulation of breast tissue^16^.

Some limitations should be taken into account when interpreting our study findings. For SIR/EAR and cumulative incidence analyses, one should be cautious with interpreting differences between categories, as there might be differences in the duration of follow-up and pelvic radiotherapy exposure between the categories. As we did not have complete data on treatments for subsequent malignant tumors before SBC (66 survivors had subsequent malignant tumors before SBC diagnosis), we were not able to explore the effects of those treatments on SBC risk. However, our sensitivity analyses censoring at the time of the first subsequent malignant tumor (Extended Data Table 5b) were consistent with the results in our main analyses. Our results of a 1.7-time increased risk of SBC compared to the general female population for survivors who received neither chest radiotherapy nor doxorubicin (Extended Data Table 2) suggest that other factors, such as genetic predisposition, may also have a role. As we had incomplete information on genetic cancer predispositions in our study, we were not able to evaluate genetic effects and possible gene–treatment interactions. The SJLIFE study, however, demonstrated that anthracycline effects are independent of cancer predisposition gene mutations^13^. Future studies with germline genetic sequencing data may help to further elucidate the interplay of genetic modifiers and individual chemotherapeutic agent exposure on SBC risk. Furthermore, we did not have information on some other SBC risk factors, such as unhealthy lifestyle (for example, low-level physical activity, obesity and excessive alcohol use) and reproductive history, which could potentially lead to some degree of confounding bias. Our analyses did not identify associations between gonadotoxic therapies (pelvic radiotherapy and alkylating agents as proxies for reproductive history) and SBC risk, making it unlikely that reproductive factors are strong confounders. Also, most lifestyle factors are not very likely to be associated with anthracycline exposure. This has also been described in a previous study showing that anthracyclines were not associated with being insufficiently active or having high-risk health behaviors^24^. Therefore, we assume that the risk of confounding caused by lifestyle factors is very low. However, it is important to evaluate this in future studies.

According to the current IGHG breast cancer screening guideline, survivors with a relative risk more than two times higher than survivors not exposed to a specific treatment are considered to be at moderate or high risk for SBC. Recommendations for SBC screening in survivors are based on these risk levels^8^. The current IGHG guideline was not able to formulate SBC screening recommendations for survivors treated with anthracyclines because there was inconsistent evidence on dose thresholds for classifying survivors as moderate or high risk and no data on possible dose–effect differences in risks for the different individual anthracycline agents. In our study, we observed a more than two times higher risk of SBC for survivors treated with ≥200 mg m^−^^2^ cumulative doxorubicin dose compared to the no doxorubicin treatment. Given that trends in childhood cancer treatments include reduced use of chest radiation therapy doses and increased exposure to anthracyclines since the 1970s^7^, our findings support that early initiation of breast cancer surveillance may be reasonable for childhood cancer survivors who have received ≥200 mg m^−^^2^ cumulative doxorubicin dose. We believe that these findings should be implemented in an update of the SBC surveillance guideline for survivors. Our study also provided insufficient information on the dose–response relation of epirubicin on SBC risk to advise on screening recommendations for this anthracycline agent.

In conclusion, doxorubicin is associated with a dose-dependent increase of SBC, both in women treated with and without chest radiotherapy. Epirubicin is also associated with an increased SBC risk. Our findings support that it may be reasonable to initiate early breast cancer screening in female childhood cancer survivors who have received ≥200 mg m^−^^2^ cumulative doxorubicin dose. We believe that the results of our study should be considered in updates of the SBC surveillance guidelines for survivors and can inform future treatment protocols for newly diagnosed childhood cancer patients.

## Methods

### Study population

We pooled data from five cohort studies (CCSS (9,671 women diagnosed in period 1970–1999), SJLIFE (2,236 women diagnosed in period 1962–2012), DCCSS-LATER (2,237 women diagnosed in period 1963–2001), FCCSS (3,415 women diagnosed in period 1943–2000) and Dutch Hodgkin Late Effects cohort (265 women diagnosed in period 1965–1995)) and one case–cohort study (SCCSS, 79 women diagnosed in period 1976–2007) in Europe and North America with available data on radiotherapy cumulative dose and fields and cumulative dose for chemotherapy (Fig. 1). Details of the study design and methodology have been previously described^25^. Briefly, eligibility criteria included a primary cancer diagnosis at <21 years of age, survival ≥5 years from primary cancer diagnosis, follow-up data on the presence and type of subsequent primary neoplasms.

### Ethics approval

The contributing cohort study teams obtained institutional review board and/or Ethics Committee approval or exemption in their respective contributing institute (CCSS: The St. Jude Children’s Research Hospital Institutional Review Board (ref. 021289), SJLIFE: The St. Jude Children’s Research Hospital Institutional Review Board (ref. 021898); DCCSS-LATER: Medical Ethical Committee Academic Medical Center, Amsterdam (ref. MEC 08/2014); FCCSS: ethics committee of the INSERM (ref. 12-077); Dutch Hodgkin Late Effects cohort: the NKI-AVL Institutional Review Board (ref. IRBd20-155); SCCSS: the cantonal ethics committee Bern (ref. KEK BE 166/2014 and KEK BE 183/11)). The pooling effort is exempt from review in compliance with Dutch law and regulations for health research involving human beings. Data sharing agreements between the Princess Máxima Center for Pediatric Oncology and all data providers are in place. Written consent was obtained from all patients of the CCSS and the SJLIFE. Specific informed consent for retrospective data collections for selected groups of patients for the DCCSS-LATER, the Dutch Hodgkin Late Effects cohort, the FCCSS and the SCCSS cohorts was waived in accordance with the country’s legislation.

### Ascertainment of treatment information and SBC diagnosis

For each patient in the individual cohorts, diagnostic information of the childhood cancer and treatment details of primary cancer and recurrences were ascertained by medical record abstraction^25^. CED was calculated and used as the cumulative exposure of alkylating agents^26^. Radiotherapy fields involving the chest, collectively referred to as ‘chest radiotherapy’ included whole lung, TBI, mantle, mediastinal and other chest-exposing fields (for example, axilla and spine). Pelvic radiotherapy included any field involving the pelvis, including TBI.

Methods for ascertainment and validation of SBC differed among the included cohorts. The study teams applied various combinations of cancer registry linkage, self-reported survey data with medical record validation for survivors who reported SBC and/or information extracted from pathology reports or medical records (Supplementary Table 6). Details regarding cohort-specific methodology for definitions of treatment exposures and subsequent tumor ascertainment were reported previously^25^. Vital status was ascertained by linkages to national death registries and/or by medical records.

### Statistical analysis

Childhood cancer survivors were considered at risk for developing SBC from 5 years after a primary cancer diagnosis until the date of the first SBC, death or the date of the last follow-up observation, whichever occurred first.

The incidence of SBC in the pooled cohort was compared with the general female population using country-specific incidence rates of the Cancer Incidence in Five Continents database (CI5, https://ci5.iarc.fr/), a database from the International Agency for Research on Cancer containing information from cancer registries worldwide^27^. Because no French nationwide incidence data were available from the CI5, we used data from the French cancer registry network Francim for the incidence rate of breast cancer in France^28–30^. SIRs were calculated as the ratio of the observed number of SBC to the expected number of female breast cancers. Expected numbers were estimated by accumulating cohort-specific person-years at risk by country, age (5-year bands), and calendar year (1-year bands)-specific strata and multiplying by the corresponding female breast cancer incidence rates in the general population. EARs were calculated as the differences between observed and expected numbers of female breast cancer per 1,000 person-years at risk. Because population-based breast cancer incidence rates only include invasive tumors, we considered the first invasive breast cancer as an event for these analyses. Cumulative incidences of SBC overall and by treatment subgroups were calculated, treating death as a competing risk.

Multivariable Cox proportional hazards regression analyses, stratified by cohort, were used to compute HRs and 95% CIs of SBC, either invasive breast cancer or DCIS, according to treatment exposure categories using a one-stage approach and stratifying the analyses by cohort. Attained age was used as the time scale^31^. Weights were applied to account for the case–cohort data from the SCCSS and for the under-sampling of acute lymphoblastic leukemia cases in the CCSS data. The proportional hazards assumption was checked with scaled Schoenfeld residuals in Cox models; it was not violated. The base multivariable model included specific anthracycline agents, age at primary cancer diagnosis, the combination of the chest radiation field and its associated maximum dose, pelvic radiation dose ≥5 Gy and alkylating agent CED exposure, all of which have been shown or suggested to be associated with breast cancer risk in previous studies^6,11,16,32,33^. We modeled cumulative doxorubicin dose and daunorubicin dose (categories by steps of 100 mg m^−2^ to ≥400 mg m^−^^2^ for doxorubicin dose and to ≥200 mg m^−^^2^ for daunorubicin dose due to statistical power reasons, respectively, and continuously per 100 mg m^−2^ increase) and epirubicin (yes/no); this proved infeasible for idarubicin owing to limited numbers of females treated with this agent. We first categorized chest radiotherapy as the combination of each eligible radiation field (defined above) with the associated maximum chest radiotherapy dose below or above the median categorized as low-dose or high-dose, respectively. Because results were comparable for fields with similar levels of potential radiation exposure to the breast, we categorized chest radiotherapy as follows: no chest radiotherapy, high-dose mantle, low-dose mantle, mediastinal, TBI, whole lung and other. Because there is only evidence for associations between anthracyclines and alkylating agents on SBC risk, we applied the following selection procedure to evaluate other chemotherapeutic agents: we added binary indicators for epipodophyllotoxins, vinca alkaloids, platinum compounds and antimetabolites to the base model. If addition of each variable changed any HRs of cumulative doxorubicin and/or daunorubicin dose by >10% compared to a model without the variable, it was included in the final models (Supplementary Table 7). Our final multivariable analyses did not include any of the additional classes of chemotherapeutic agents indicated above.

Interaction between cumulative doxorubicin/daunorubicin doses and chest radiotherapy and age at primary childhood cancer diagnosis on a multiplicative scale was evaluated by comparing models with and without the interaction term via likelihood ratio tests. Aalen’s additive hazard models were applied to evaluate the interaction of cumulative doxorubicin/daunorubicin exposures and chest radiotherapy and age at primary childhood cancer diagnosis on an additive scale^34^.

A series of prespecified sensitivity analyses were conducted by applying the same regression models to the data with (a) outcome restricted to invasive breast cancer to exclude DCIS, which does not always progress to invasive breast cancer; (b) censoring at the time of the first non-SBC subsequent malignant neoplasm diagnosis to rule out effects of treatments for those tumors; (c) excluding 444 survivors treated for childhood cancer before 1970 to exclude a potentially influential group of women who reached comparatively high attained age yet showing deviating characteristics owing to improvements in clinical practice and survival trends since the 1970s; (d) excluding survivors treated for Hodgkin lymphoma to exclude patients who generally received extensive radiotherapy fields to the chest (Extended Data Table 5) (e) excluding each cohort on a one-by-one basis to evaluate robustness of findings (Supplementary Table 4) and each cohort to evaluate between-cohort differences (Supplementary Table 5).

All analyses were conducted in R software (version 4.0.3). A *P* value of <0.05 was considered statistically significant in two-sided statistical tests.

### Reporting summary

Further information on research design is available in the Nature Portfolio Reporting Summary linked to this article.

## Online content

Any methods, additional references, Nature Portfolio reporting summaries, source data, extended data, supplementary information, acknowledgements, peer review information; details of author contributions and competing interests; and statements of data and code availability are available at 10.1038/s41591-023-02514-1.

## Supplementary information

Supplementary Information Supplementary Tables 1–7.

Reporting Summary

## Figures and Tables

**Extended Data Table 1:**
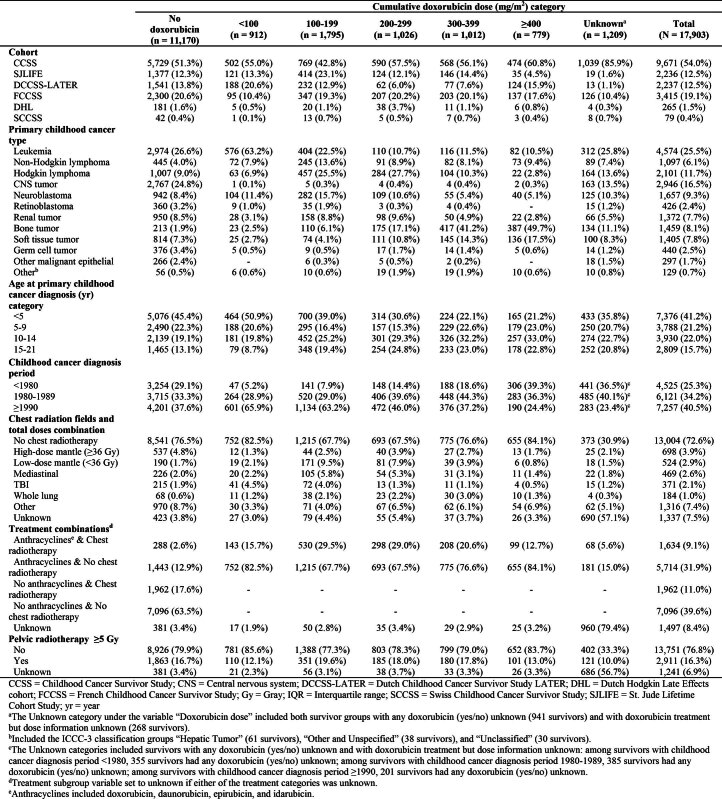
Demographic and treatment characteristics of female five-year childhood cancer survivors by cumulative doxorubicin dose categories

**Extended Data Table 2:**
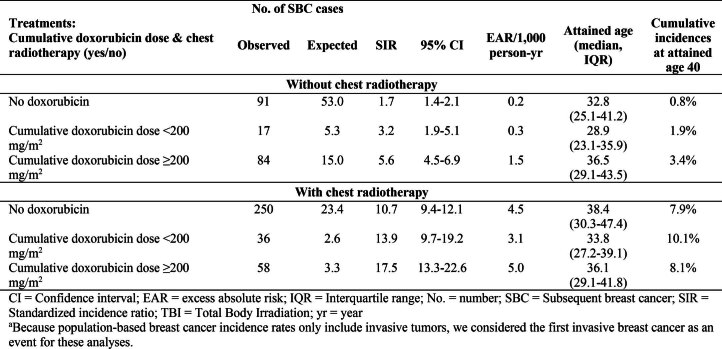
Standardized incidence ratios, excess absolute risks, and cumulative incidences by treatments^a^

**Extended Data Table 3:**
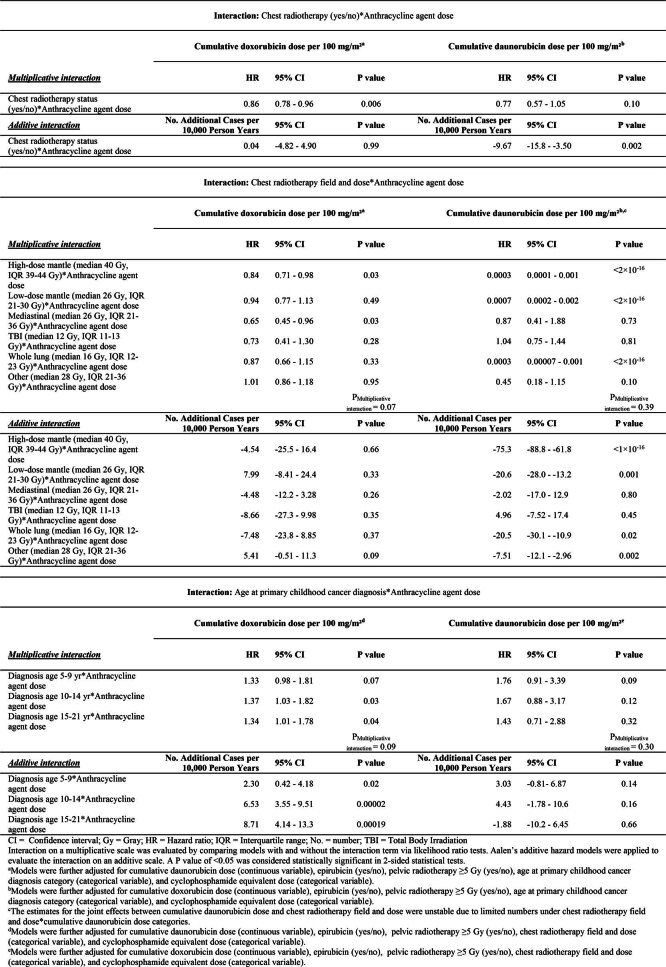
Joint effects of cumulative doxorubicin and daunorubicin doses and other relevant risk factors on subsequent breast cancer among female five-year childhood cancer survivors

**Extended Data Table 4:**
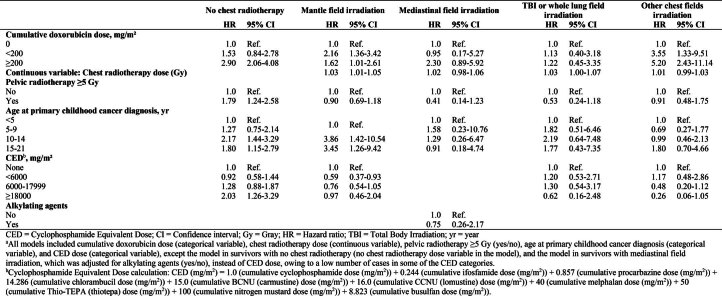
Multivariable Cox proportional hazard regression analyses for subsequent breast cancer in female five-year childhood cancer survivors with different chest radiotherapy fields irradiation^a^

**Extended Data Table 5:**
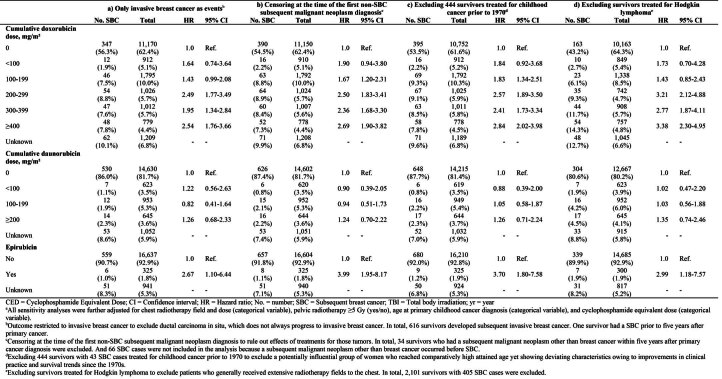
Sensitivity analyses for risk of subsequent breast cancer on subgroups with different treatments or events^a^

**Extended Data Table 6:**
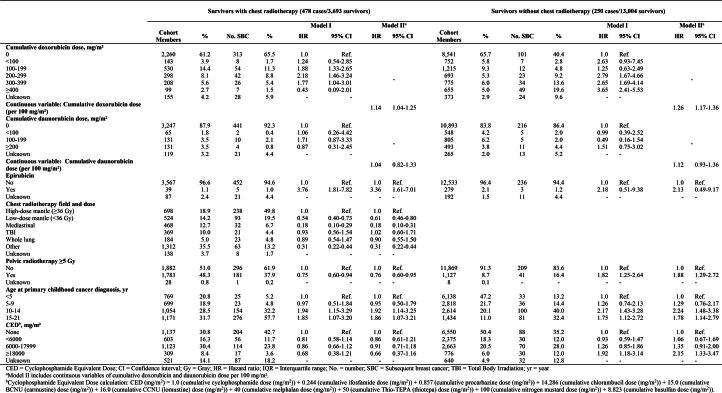
Multivariable Cox proportional hazard regression analyses for subsequent breast cancer in female five-year childhood cancer survivors stratified by chest radiotherapy status (yes/no)

## Data Availability

The International Consortium for Pooled Studies on Subsequent Malignancies after Childhood and Adolescent Cancer database is not an open-access database due to ethical and data protection constraints. The pseudonymized data are managed by the Princess Máxima Center for Pediatric Oncology in the Netherlands and cannot be shared with investigators outside the institute without consent from all involved parties. However, potential collaborators are welcome to submit proposals to J.C.T. (j.c.teepen@prinsesmaximacentrum.nl), which will be considered by the consortium. The consortium will come up with a decision about submitted proposals within 3 months after application. The country-specific female breast cancer rates of the Cancer Incidence in Five Continents database were used as the general female population in our study. These data are publicly available: https://ci5.iarc.fr/.

## Code availability

The statistical code for our main analyses is available at https://github.com/yuehanwanggitub/IPDanthracyclines.git.
