# Supplementary material for: Subsequent female breast cancer risk associated with anthracycline chemotherapy for childhood cancer
Source: Nat Med. 2023 Sep 11;29(9):2268–77. doi: 10.1038/s41591-023-02514-1 (PMC10504074; doi:10.1038/s41591-023-02514-1)
Supplement: Supplementary file 1 — Supplementary Tables 1–7. [file 41591_2023_2514_MOESM1_ESM.pdf]

nature medicine

Article

https://doi.org/10.1038/s41591-023-02514-1

Subsequent female breast cancer risk associated with anthracycline chemotherapy for childhood cancer

In the format provided by the authors and unedited

Supplementary Table 1 | Demographic and treatment characteristics of 17,903 female five-year childhood cancer survivors (primary cancer diagnosis year 1946-2012) overall and by subsequent breast cancer status

	Total (n = 17,903)	Subsequent breast cancer[a] (n = 782)	No subsequent breast cancer (n = 17,121)
	**No. (%)**	**No. (%)**	**No. (%)**
**Primary childhood cancer[b]**			
Leukemia	4,574 (25.5)	81 (10.4)	4,493 (26.2)
Non-Hodgkin lymphoma	1,097 (6.1)	37 (4.7)	1,060 (6.2)
Hodgkin lymphoma	2,101 (11.7)	405 (51.8)	1,696 (9.9)
Central nervous system tumor	2,946 (16.5)	14 (1.8)	2,932 (17.1)
Neuroblastoma	1,657 (9.3)	15 (1.9)	1,642 (9.6)
Retinoblastoma	426 (2.4)	2 (0.3)	424 (2.5)
Renal tumor	1,372 (7.7)	45 (5.8)	1,327 (7.8)
Bone tumor	1,459 (8.1)	106 (13.6)	1,353 (7.9)
Soft tissue tumor	1,405 (7.8)	55 (7.0)	1,350 (7.9)
Germ cell tumor	440 (2.5)	9 (1.2)	431 (2.5)
Other malignant epithelial	297 (1.7)	11 (1.4)	286 (1.7)
Other[c]	129 (0.7)	2 (0.2)	127 (0.8)
**Cumulative doxorubicin dose, mg/m^2**			
0	11,170 (62.4)	431 (55.1)	10,739 (62.7)
<100	912 (5.1)	16 (2.0)	896 (5.2)
100-199	1,795 (10.0)	69 (8.8)	1,726 (10.1)
200-299	1,026 (5.7)	67 (8.6)	959 (5.6)
300-399	1,012 (5.7)	64 (8.2)	948 (5.5)
≥400	779 (4.4)	58 (7.4)	721 (4.2)
Unknown[d]	1,209 (6.8)	77 (9.8)	1,132 (6.6)
**Cumulative daunorubicin dose, mg/m^2**			
0	14,630 (81.7)	684 (87.5)	13,946 (81.5)
<100	623 (3.5)	7 (0.9)	616 (3.6)
100-199	953 (5.3)	16 (2.0)	937 (5.5)
≥200	645 (3.6)	17 (2.2)	628 (3.7)
Unknown[e]	1,052 (5.9)	58 (7.4)	994 (5.8)
**Epirubicin**			
No	16,637 (92.9)	717 (91.7)	15,920 (93.0)
Yes	325 (1.8)	9 (1.2)	316 (1.8)
Unknown	941 (5.3)	56 (7.2)	885 (5.2)
**Idarubicin**			
No	16,843 (94.1)	725 (92.7)	16,118 (94.1)
Yes	107 (0.6)	1 (0.1)	106 (0.6)
Unknown	953 (5.3)	56 (7.2)	897 (5.2)
**CED[f]**			
0	7,951 (44.4)	301 (38.5)	7,650 (44.7)
<6000	3,069 (17.1)	94 (12.0)	2,975 (17.4)
6000-17999	3,899 (21.8)	192 (24.6)	3,707 (21.7)
≥18000	1,117 (6.2)	47 (6.0)	1,070 (6.2)
Unknown	1,867 (10.4)	148 (18.9)	1,719 (10.0)

Supplementary Table 1 | Demographic and treatment characteristics of 17,903 female five-year childhood cancer survivors (primary cancer diagnosis year 1946-2012) overall and by subsequent breast cancer status

	Total (n = 17,903)	Subsequent breast cancer[a] (n = 782)	No subsequent breast cancer (n = 17,121)
	No. (%)	No. (%)	No. (%)
**Epipodophyllotoxins**			
No	13,434 (75.0)	611 (78.1)	12,823 (74.9)
Yes	3,346 (18.7)	78 (10.0)	3,268 (19.1)
Unknown	1,123 (6.3)	93 (11.9)	1,030 (6.0)
**Vinca Alkaloids**			
No	6,194 (34.6)	269 (34.4)	5,925 (34.6)
Yes	10,586 (59.1)	420 (53.7)	10,166 (59.4)
Unknown	1,123 (6.3)	93 (11.9)	1,030 (6.0)
**Platinum Compounds**			
No	14,219 (79.4)	642 (82.1)	13,577 (79.3)
Yes	2,561 (14.3)	47 (6.0)	2,514 (14.7)
Unknown	1,123 (6.3)	93 (11.9)	1,030 (6.0)
**Antimetabolites**			
No	10,376 (58.0)	504 (64.5)	9,872 (57.7)
Yes	6,404 (35.8)	185 (23.7)	6,219 (36.3)
Unknown	1,123 (6.3)	93 (11.9)	1,030 (6.0)
**Chest radiotherapy fields and doses[g]**			
No chest radiotherapy	13,004 (72.6)	250 (32.0)	12,754 (74.5)
High-dose mantle (≥36 Gy) median 40 Gy, IQR 39-44 Gy[h]	698 (3.9)	238 (30.4)	460 (2.7)
Low-dose mantle (<36 Gy) median 26 Gy, IQR 21-30 Gy[h]	524 (2.9)	93 (11.9)	431 (2.5)
Mediastinal median 26 Gy, IQR 21-36 Gy[h]	469 (2.6)	33 (4.2)	436 (2.5)
TBI median 12 Gy, IQR 11-13 Gy[h]	371 (2.1)	22 (2.8)	349 (2.0)
Whole lung median 16 Gy, IQR 12-23 Gy[h]	184 (1.0)	23 (2.9)	161 (0.9)
Other median 28 Gy, IQR 21-36 Gy[h]	1,316 (7.4)	63 (8.1)	1,253 (7.3)
Unknown	1,337 (7.5)	60 (7.7)	1,277 (7.5)
**Pelvic radiotherapy dose[i]**			
No pelvic radiotherapy	13,727 (76.7)	505 (64.6)	13,222 (77.2)
<10 Gy	142 (0.8)	6 (0.8)	136 (0.8)
10-19 Gy	594 (3.3)	24 (3.1)	570 (3.3)
20-29 Gy	719 (4.0)	38 (4.9)	681 (4.0)
30-39 Gy	767 (4.3)	82 (10.5)	685 (4.0)
≥40 Gy	713 (4.0)	72 (9.2)	641 (3.7)
Unknown	1,241 (6.9)	55 (7.0)	1,186 (6.9)
**Age at diagnosis of primary cancer, yrs**			
<5	7,376 (41.2)	66 (8.4)	7,310 (42.7)
5-9	3,788 (21.2)	65 (8.3)	3,723 (21.7)
10-14	3,930 (22.0)	273 (34.9)	3,657 (21.4)
15-21	2,809 (15.7)	378 (48.3)	2,431 (14.2)
**Treatment subgroups[j]**			
Anthracycline[k] & Chest radiotherapy	1,634 (9.1)	163 (20.8)	1,471 (8.6)
Anthracycline & No Chest radiotherapy	5,714 (31.9)	156 (19.9)	5,558 (32.5)

Supplementary Table 1 | Demographic and treatment characteristics of 17,903 female five-year childhood cancer survivors (primary cancer diagnosis year 1946-2012) overall and by subsequent breast cancer status

	**Total (n = 17,903)**	**Subsequent breast cancer[a] (n = 782)**	**No subsequent breast cancer (n = 17,121)**
	**No. (%)**	**No. (%)**	**No. (%)**
No Anthracycline & Chest radiotherapy	1,962 (11.0)	294 (37.6)	1,668 (9.7)
No Anthracycline & No Chest radiotherapy	7,096 (39.6)	83 (10.6)	7,013 (41.0)
Unknown	1,497 (8.4)	86 (11.0)	1,411 (8.2)
**Period of primary cancer diagnosis**			
<1960	60 (0.3)	9 (1.2)	51 (0.3)
1960-1969	384 (2.1)	34 (4.3)	350 (2.0)
1970-1979	4,081 (22.8)	343 (43.9)	3,738 (21.8)
1980-1989	6,121 (34.2)	283 (36.2)	5,838 (34.1)
1990-1999	6,134 (34.3)	107 (13.7)	6,027 (35.2)
2000-2012	1,123 (6.3)	6 (0.8)	1,117 (6.5)
**Time since five-year of primary cancer diagnosis, yrs**			
<10	2,448 (13.7)	64 (8.2)	2,384 (13.9)
10-19	6,504 (36.3)	310 (39.6)	6,194 (36.2)
20-29	5,332 (29.8)	315 (40.3)	5,017 (29.3)
≥30	3,619 (20.2)	93 (11.9)	3,526 (20.6)
**Attained age, yrs**			
<20	1,870 (10.4)	3 (0.4)	1,867 (10.9)
20-29	4,882 (27.3)	76 (9.7)	4,806 (28.1)
30-39	5,847 (32.7)	322 (41.2)	5,525 (32.3)
≥40	5,304 (29.6)	381 (48.7)	4,923 (28.8)
**Vital status**			
Alive at last contact	15,278 (85.3)	549 (70.2)	14,729 (86.0)
Deceased at last contact	2,625 (14.7)	233 (29.8)	2,392 (14.0)

CED = Cyclophosphamide Equivalent Dose; Gy = Gray; IQR = Interquartile range; No. = number; TBI = Total Body Irradiation; yr = year

[a]Included both invasive and ductal carcinoma in situ breast cancer.

[b]Because of eligibility criteria of the cohort, the composition of primary cancer diagnosis groups in our pooled data may differ from the composition in underlying populations of childhood cancer survivors.

[c]Included the ICCC-3 classification groups "Hepatic Tumor" (0 case/61 survivors), "Other and Unspecified" (1 case/38 survivors), and "Unclassified" (1 case/30 survivors).

[d]The Unknown category under the variable "Doxorubicin dose" included both survivor groups with any doxorubicin (yes/no) unknown (56 cases/941 survivors) and with doxorubicin treatment but dose information unknown (21 cases/268 survivors).

[e]The Unknown category under the variable "Daunorubicin dose" included both survivor groups with any daunorubicin (yes/no) unknown (56 cases/953 survivors) and with daunorubicin treatment but dose information unknown (2 cases/99 survivors).

[f]Cyclophosphamide Equivalent Dose calculation: CED (mg/m^2) = 1.0 (cumulative cyclophosphamide dose (mg/m^2)) + 0.244 (cumulative ifosfamide dose (mg/m^2)) + 0.857 (cumulative procarbazine dose (mg/m^2)) + 14.286 (cumulative chlorambucil dose (mg/m^2)) + 15.0 (cumulative BCNU (carmustine) dose (mg/m^2)) + 16.0 (cumulative CCNU (lomustine) dose (mg/m^2)) + 40 (cumulative melphalan dose (mg/m^2)) + 50 (cumulative Thio-TEPA (thiotepa) dose (mg/m^2)) + 100 (cumulative nitrogen mustard dose (mg/m^2)) + 8.823 (cumulative busulfan dose (mg/m^2)).

[g]Included radiotherapy fields exposing (parts of) the chest. Radiation dose refered to the cumulative prescribed dose (including boost doses, if applicable), or slight variations, depending on definitions in the underlying cohorts (see Wang et al. 2022). Chest radiotherapy was categorized as the combination of chest radiation fields with the associated maximum chest radiotherapy dose below or above the median. The variable was classified as follows: high-dose mantle (median 40 Gy, IQR 39-44 Gy), low-dose mantle (median 26 Gy, IQR 21-30 Gy), mediastinal (median 26 Gy, IQR 21-36 Gy), TBI (median 12 Gy, IQR 11-13 Gy), whole lung (median 16 Gy, IQR 12-23 Gy), other (median 28 Gy, IQR 21-36 Gy), and unknown.

[h]Dose represents the maximum cumulative prescribed chest dose (including boost doses, if applicable) of survivors classified in this group. This could include doses to chest field other than this category.

[i]Included radiotherapy fields exposing (parts of) the pelvis (including TBI). Radiation dose refered to the cumulative prescribed dose (including boost doses, if applicable), or slight variations, depending on definitions in the underlying cohorts (see Wang et al. 2022). The Unknown category under the variable "Pelvic radiotherapy dose" included both survivor groups with any pelvic radiotherapy (yes/no) unknown (54 cases/1,212 survivors) and with pelvic radiotherapy treatment but dose information unknown (1 cases/29 survivors).
[j]Treatment subgroup variable set to unknown if either of the treatment categories was unknown.
[k]Anthracyclines included doxorubicin, daunorubicin, epirubicin, and idarubicin.

Supplementary Table 2 | Demographic and treatment characteristics of 17,903 female five-year childhood cancer survivors (primary cancer diagnosis year 1946-2012) overall and by subsequent breast cancer status (row percentage)

	Total (n = 17,903)	Subsequent breast cancer[a] (n = 782)	No subsequent breast cancer (n = 17,121)
	No.	No. (%)	No. (%)
**Primary childhood cancer[b]**			
Leukemia	4,574	81 (1.8)	4,493 (98.2)
Non-Hodgkin lymphoma	1,097	37 (3.4)	1,060 (96.6)
Hodgkin lymphoma	2,101	405 (19.3)	1,696 (80.7)
Central nervous system tumor	2,946	14 (0.5)	2,932 (99.5)
Neuroblastoma	1,657	15 (0.9)	1,642 (99.1)
Retinoblastoma	426	2 (0.5)	424 (99.5)
Renal tumor	1,372	45 (3.3)	1,327 (96.7)
Bone tumor	1,459	106 (7.3)	1,353 (92.7)
Soft tissue tumor	1,405	55 (3.9)	1,350 (96.1)
Germ cell tumor	440	9 (2.0)	431 (98.0)
Other malignant epithelial	297	11 (3.7)	286 (96.3)
Other[c]	129	2 (1.6)	127 (98.4)
**Cumulative doxorubicin dose, mg/m^2**			
0	11,170	431 (3.9)	10,739 (96.1)
<100	912	16 (1.8)	896 (98.2)
100-199	1,795	69 (3.8)	1,726 (96.2)
200-299	1,026	67 (6.5)	959 (93.5)
300-399	1,012	64 (6.3)	948 (93.7)
≥400	779	58 (7.4)	721 (92.6)
Unknown[d]	1,209	77 (6.4)	1,132 (93.6)
**Cumulative daunorubicin dose, mg/m^2**			
0	14,630	684 (4.7)	13,946 (95.3)
<100	623	7 (1.1)	616 (98.9)
100-199	953	16 (1.7)	937 (98.3)
≥200	645	17 (2.6)	628 (97.4)
Unknown[e]	1,052	58 (5.5)	994 (94.5)
**Epirubicin**			
No	16,637	717 (4.3)	15,920 (95.7)
Yes	325	9 (2.8)	316 (97.2)
Unknown	941	56 (6.0)	885 (94.0)
**Idarubicin**			
No	16,843	725 (4.3)	16,118 (95.7)
Yes	107	1 (0.9)	106 (99.1)
Unknown	953	56 (5.9)	897 (94.1)
**CED[f]**			
0	7,951	301 (3.8)	7,650 (96.2)
<6000	3,069	94 (3.1)	2,975 (96.9)
6000-17999	3,899	192 (4.9)	3,707 (96.9)
≥18000	1,117	47 (4.2)	1,070 (95.8)
Unknown	1,867	148 (7.9)	1,719 (92.1)

Supplementary Table 2 | Demographic and treatment characteristics of 17,903 female five-year childhood cancer survivors (primary cancer diagnosis year 1946-2012) overall and by subsequent breast cancer status (row percentage)

	Total (n = 17,903)	Subsequent breast cancer[a] (n = 782)	No subsequent breast cancer (n = 17,121)
	**No.**	**No. (%)**	**No. (%)**
**Epipodophyllotoxins**			
No	13,434	611 (4.5)	12,823 (95.5)
Yes	3,346	78 (2.3)	3,268 (97.7)
Unknown	1,123	93 (8.3)	1,030 (91.7)
**Vinca Alkaloids**			
No	6,194	269 (4.3)	5,925 (95.7)
Yes	10,586	420 (4.0)	10,166 (96.0)
Unknown	1,123	93 (8.3)	1,030 (91.7)
**Platinum Compounds**			
No	14,219	642 (4.5)	13,577 (95.5)
Yes	2,561	47 (1.8)	2,514 (98.2)
Unknown	1,123	93 (8.3)	1,030 (91.7)
**Antimetabolites**			
No	10,376	504 (4.9)	9,872 (95.1)
Yes	6,404	185 (2.9)	6,219 (97.1)
Unknown	1,123	93 (8.3)	1,030 (91.7)
**Chest radiotherapy fields and doses[g]**			
No chest radiotherapy	13,004	250 (1.9)	12,754 (98.1)
High-dose mantle (≥36 Gy) median 40 Gy, IQR 39-44 Gy[h]	698	238 (34.1)	460 (65.9)
Low-dose mantle (<36 Gy) median 26 Gy, IQR 21-30 Gy[h]	524	93 (17.7)	431 (82.3)
Mediastinal median 26 Gy, IQR 21-36 Gy[h]	469	33 (7.0)	436 (93.0)
TBI median 12 Gy, IQR 11-13 Gy[h]	371	22 (5.9)	349 (94.1)
Whole lung median 16 Gy, IQR 12-23 Gy[h]	184	23 (12.5)	161 (87.5)
Other median 28 Gy, IQR 21-36 Gy[h]	1,316	63 (4.8)	1,253 (95.2)
Unknown	1,337	60 (4.5)	1,277 (95.5)
**Pelvic radiotherapy dose[i]**			
No pelvic radiotherapy	13,727	505 (3.7)	13,222 (96.3)
<10 Gy	142	6 (4.2)	136 (95.8)
10-19 Gy	594	24 (4.0)	570 (96.0)
20-29 Gy	719	38 (5.3)	681 (94.7)
30-39 Gy	767	82 (10.7)	685 (89.3)
≥40 Gy	713	72 (10.1)	641 (89.9)
Unknown	1,241	55 (4.4)	1,186 (95.6)
**Age at diagnosis of primary cancer, yrs**			
<5	7,376	66 (0.9)	7,310 (99.1)
5-9	3,788	65 (1.7)	3,723 (98.3)
10-14	3,930	273 (6.9)	3,657 (93.1)
15-21	2,809	378 (13.5)	2,431 (86.5)
**Treatment subgroups[j]**			
Anthracycline[k] & Chest radiotherapy	1,634	163 (10.0)	1,471 (90.0)
Anthracycline & No Chest radiotherapy	5,714	156 (2.7)	5,558 (97.3)

Supplementary Table 2 | Demographic and treatment characteristics of 17,903 female five-year childhood cancer survivors (primary cancer diagnosis year 1946-2012) overall and by subsequent breast cancer status (row percentage)

	Total (n = 17,903)	Subsequent breast cancer[a] (n = 782)	No subsequent breast cancer (n = 17,121)
	No.	No. (%)	No. (%)
No Anthracycline & Chest radiotherapy	1,962	294 (15.0)	1,668 (85.0)
No Anthracycline & No Chest radiotherapy	7,096	83 (1.2)	7,013 (98.8)
Unknown	1,497	86 (5.7)	1,411 (94.3)
**Period of primary cancer diagnosis**			
<1960	60	9 (15.0)	51 (85.0)
1960-1969	384	34 (8.9)	350 (91.1)
1970-1979	4,081	343 (8.4)	3,738 (91.6)
1980-1989	6,121	283 (4.6)	5,838 (95.4)
1990-1999	6,134	107 (1.7)	6,027 (98.3)
2000-2012	1,123	6 (0.5)	1,117 (99.5)
**Time since five-year of primary cancer diagnosis, yrs**			
<10	2,448	64 (2.6)	2,384 (97.4)
10-19	6,504	310 (4.8)	6,194 (95.2)
20-29	5,332	315 (5.9)	5,017 (94.1)
≥30	3,619	93 (2.6)	3,526 (97.4)
**Attained age, yrs**			
<20	1,870	3 (0.2)	1,867 (99.8)
20-29	4,882	76 (1.6)	4,806 (98.4)
30-39	5,847	322 (5.5)	5,525 (94.5)
≥40	5,304	381 (7.2)	4,923 (92.8)
**Vital status**			
Alive at last contact	15,278	549 (3.6)	14,729 (96.4)
Deceased at last contact	2,625	233 (8.9)	2,392 (91.1)

CED = Cyclophosphamide Equivalent Dose; Gy = Gray; IQR = Interquartile range; No. = number; TBI = Total Body Irradiation; yr = year

[a]Included both invasive and ductal carcinoma in situ breast cancer.

[b]Because of eligibility criteria of the cohort, the composition of primary cancer diagnosis groups in our pooled data may differ from the composition in underlying populations of childhood cancer survivors.

[c]Included the ICCC-3 classification groups "Hepatic Tumor" (0 case/61 survivors), "Other and Unspecified" (1 case/38 survivors), and "Unclassified" (1 case/30 survivors).

[d]The Unknown category under the variable "Doxorubicin dose" included both survivor groups with any doxorubicin (yes/no) unknown (56 cases/941 survivors) and with doxorubicin treatment but dose information unknown (21 cases/268 survivors).

[e]The Unknown category under the variable "Daunorubicin dose" included both survivor groups with any daunorubicin (yes/no) unknown (56 cases/953 survivors) and with daunorubicin treatment but dose information unknown (2 cases/99 survivors).

[f]Cyclophosphamide Equivalent Dose calculation: CED (mg/m^2) = 1.0 (cumulative cyclophosphamide dose (mg/m^2)) + 0.244 (cumulative ifosfamide dose (mg/m^2)) + 0.857 (cumulative procarbazine dose (mg/m^2)) + 14.286 (cumulative chlorambucil dose (mg/m^2)) + 15.0 (cumulative BCNU (carmustine) dose (mg/m^2)) + 16.0 (cumulative CCNU (lomustine) dose (mg/m^2)) + 40 (cumulative melphalan dose (mg/m^2)) + 50 (cumulative Thio-TEPA (thiotepa) dose (mg/m^2)) + 100 (cumulative nitrogen mustard dose (mg/m^2)) + 8.823 (cumulative busulfan dose (mg/m^2)).

[g]Included radiotherapy fields exposing (parts of) the chest. Radiation dose refered to the cumulative prescribed dose (including boost doses, if applicable), or slight variations, depending on definitions in the underlying cohorts (see Wang et al. 2022). Chest radiotherapy was categorized as the combination of chest radiation fields with the associated maximum chest radiotherapy dose below or above the median. The variable was classified as follows: high-dose mantle (median 40 Gy, IQR 39-44 Gy), low-dose mantle (median 26 Gy, IQR 21-30 Gy), mediastinal (median 26 Gy, IQR 21-36 Gy), TBI (median 12 Gy, IQR 11-13 Gy), whole lung (median 16 Gy, IQR 12-23 Gy), other (median 28 Gy, IQR 21-36 Gy), and unknown.

[h]Dose represents the maximum cumulative prescribed chest dose (including boost doses, if applicable) of survivors classified in this group. This could include doses to chest field other than this category.

[i]Included radiotherapy fields exposing (parts of) the pelvis (including TBI). Radiation dose refered to the cumulative prescribed dose (including boost doses, if applicable), or slight variations, depending on definitions in the underlying cohorts (see Wang et al. 2022). The Unknown category under the variable "Pelvic radiotherapy dose" included both survivor groups with any pelvic radiotherapy (yes/no) unknown (54 cases/1,212 survivors) and with pelvic radiotherapy treatment but dose information unknown (1 cases/29 survivors).
[j]Treatment subgroup variable set to unknown if either of the treatment categories was unknown.
[k]Anthracyclines included doxorubicin, daunorubicin, epirubicin, and idarubicin.

Supplementary Table 3 | Demographic and treatment characteristics of female five-year childhood cancer survivors by cohort

	CCSS (n = 9,671)	SJLIFE (n = 2,236)	DCCSS-LATER (n = 2,237)	FCCSS (n = 3,415)	DHL (n = 265)	SCCSS (n = 79)	Total (N = 17,903)
**Primary childhood cancer type**							
Leukemia	2,987 (30.9%)	802 (35.9%)	770 (34.4%)	-	-	15 (19.0%)	4,574 (25.5%)
Non-Hodgkin lymphoma	586 (6.1%)	115 (5.1%)	157 (7.0%)	235 (6.9%)	-	4 (5.1%)	1,097 (6.1%)
Hodgkin lymphoma	1,276 (13.2%)	227 (10.2%)	125 (5.6%)	189 (5.5%)	265 (100%)	19 (24.1%)	2,101 (11.7%)
Central nervous system tumor	1,841 (19.0%)	287 (12.8%)	312 (13.9%)	498 (14.6%)	-	8 (10.1%)	2,946 (16.5%)
Neuroblastoma	901 (9.3%)	101 (4.5%)	145 (6.5%)	505 (14.8%)	-	5 (6.3%)	1,657 (9.3%)
Retinoblastoma	-	119 (5.3%)	14 (0.6%)	293 (8.6%)	-	-	426 (2.4%)
Renal tumor	389 (4.0%)	170 (7.6%)	250 (11.2%)	558 (16.3%)	-	5 (6.3%)	1,372 (7.7%)
Bone tumor	884 (9.1%)	133 (5.9%)	141 (6.3%)	295 (8.6%)	-	6 (7.6%)	1,459 (8.1%)
Soft tissue tumor	763 (7.9%)	127 (5.7%)	151 (6.8%)	361 (10.6%)	-	3 (3.8%)	1,405 (7.8%)
Germ cell tumor	20 (0.2%)	68 (3.0%)	101 (4.5%)	249 (7.3%)	-	2 (2.5%)	440 (2.5%)
Other malignant epithelial	-	49 (2.2%)	50 (2.2%)	187 (5.5%)	-	11 (13.9%)	297 (1.7%)
Other[a]	24 (0.2%)	38 (1.7%)	21 (0.9%)	45 (1.3%)	-	1 (1.3%)	129 (0.7%)
**Age at primary childhood cancer diagnosis (yr)**							
Median [IQR]	7.5 [3.1, 13.7]	6.2 [2.7, 12.6]	5.4 [2.7, 10.7]	5.2 [1.7, 11.4]	18.3 [16.6, 19.7]	14.2 [6.0, 17.3]	6.7 [2.8, 13.0]
**Age at primary childhood cancer diagnosis (yr) category**							
<5	3,666 (37.9%)	973 (43.5%)	1,049 (46.9%)	1,671 (48.9%)	-	17 (21.5%)	7,376 (41.2%)
5-9	2,027 (21.0%)	468 (20.9%)	569 (25.4%)	707 (20.7%)	7 (2.6%)	10 (12.7%)	3,788 (21.2%)
10-14	2,204 (22.8%)	472 (21.1%)	471 (21.1%)	744 (21.8%)	21 (7.9%)	18 (22.8%)	3,930 (22.0%)
15-21	1,774 (18.3%)	323 (14.4%)	148 (6.6%)	293 (8.6%)	237 (89.4%)	34 (43.0%)	2,809 (15.7%)
**Period of childhood cancer diagnosis, range**							
Median [IQR]	1985 [1979, 1992]	1994 [1984, 2002]	1989 [1981, 1996]	1986 [1978, 1994]	1982 [1974, 1991]	1990 [1984, 1999]	1986 [1979, 1994]
**Period of childhood cancer diagnosis category**							
<1960	-	-	-	60 (1.8%)	-	-	60 (0.3%)
1960-1969	-	42 (1.9%)	49 (2.2%)	264 (7.7%)	29 (10.9%)	-	384 (2.1%)
1970-1979	2,639 (27.3%)	274 (12.3%)	386 (17.3%)	693 (20.3%)	81 (30.6%)	8 (10.1%)	4,081 (22.8%)
1980-1989	3,737 (38.6%)	535 (23.9%)	711 (31.8%)	1,035 (30.3%)	76 (28.7%)	27 (34.2%)	6,121 (34.2%)
1990-1999	3,295 (34.1%)	633 (28.3%)	871 (38.9%)	1,233 (36.1%)	76 (28.7%)	26 (32.9%)	6,134 (34.3%)
2000-2012	-	752 (33.6%)	220 (9.8%)	130 (3.8%)	3 (1.1%)	18 (22.8%)	1,123 (6.3%)
**Duration of follow-up since 5-yr survival and the end of follow-up[b] (yr)**							
Median [IQR]	20.2 [14.7, 28.0]	18.0 [10.3, 27.5]	16.8 [10.8, 25.0]	23.2 [16.3, 31.8]	17.6 [12.3, 25.7]	11.0 [6.7, 18.7]	19.9 [14.1, 28.2]
**Duration of follow-up since 5-yr survival and the end of follow-up[b] (yr) category**							
<10	1,096 (11.3%)	543 (24.3%)	482 (21.5%)	251 (7.4%)	40 (15.1%)	36 (45.6%)	2,448 (13.7%)
10-19	3,672 (38.0%)	703 (31.4%)	859 (38.4%)	1,130 (33.1%)	116 (43.8%)	24 (30.4%)	6,504 (36.3%)
20-29	3,012 (31.1%)	570 (25.5%)	645 (28.8%)	1,018 (29.8%)	69 (26.0%)	18 (22.8%)	5,332 (29.8%)
≥30	1,891 (19.6%)	420 (18.8%)	251 (11.2%)	1,016 (29.8%)	40 (15.1%)	1 (1.3%)	3,619 (20.2%)
**Attained age at last follow-up[b] (yr)**							

Supplementary Table 3 | Demographic and treatment characteristics of female five-year childhood cancer survivors by cohort

Median [IQR]	34.4 [26.7, 42.0]	31.8 [23.7, 39.9]	29.3 [22.1, 36.9]	35.8 [27.3, 44.0]	40.9 [35.5, 48.8]	28.6 [24.3, 37.6]	33.7 [25.9, 41.6]
**Attained age at last follow-up[b] age (yr) category**							
<20	838 (8.7%)	380 (17.0%)	395 (17.7%)	242 (7.1%)	1 (0.4%)	14 (17.7%)	1,870 (10.4%)
20-29	2,552 (26.4%)	614 (27.5%)	798 (35.7%)	862 (25.2%)	26 (9.8%)	30 (38.0%)	4,882 (27.3%)
30-39	3,314 (34.3%)	688 (30.8%)	666 (29.8%)	1,068 (31.3%)	93 (35.1%)	18 (22.8%)	5,847 (32.7%)
≥40	2,967 (30.7%)	554 (24.8%)	378 (16.9%)	1,243 (36.4%)	145 (54.7%)	17 (21.5%)	5,304 (29.6%)
**SBC[c]**							
No	9,219 (95.3%)	2,159 (96.6%)	2,196 (98.2%)	3,288 (96.3%)	200 (75.5%)	59 (74.7%)	17,121 (95.6%)
Yes	452 (4.7%)	77 (3.4%)	41 (1.8%)	127 (3.7%)	65 (24.5%)	20 (25.3%)	782 (4.4%)
**First SBC type**							
Invasive	344 (3.6%)	54 (2.4%)	36 (1.6%)	111 (3.3%)	51 (19.2%)	20 (25.3%)	616 (3.4%)
DCIS	108 (1.1%)	23 (1.0%)	5 (0.2%)	16 (0.5%)	14 (5.3%)	-	166 (0.9%)
**Vital status**							
Alive at last contact	8,174 (84.5%)	2,171 (97.1%)	1,928 (86.2%)	2,759 (80.8%)	178 (67.2%)	68 (86.1%)	15,278 (85.3%)
Deceased at last contact	1,497 (15.5%)	65 (2.9%)	309 (13.8%)	656 (19.2%)	87 (32.8%)	11 (13.9%)	2,625 (14.7%)
**Radiotherapy exposure to the chest**							
No	6,607 (68.3%)	1,706 (76.3%)	1,892 (84.6%)	2,728 (79.9%)	22 (8.3%)	49 (62.0%)	13,004 (72.6%)
Yes	2,098 (21.7%)	506 (22.6%)	341 (15.2%)	482 (14.1%)	243 (91.7%)	23 (29.1%)	3,693 (20.6%)
Unknown	966 (10.0%)	24 (1.1%)	4 (0.2%)	205 (6.0%)	-	7 (8.9%)	1,206 (6.7%)
**Chest radiation dose (Gy)**							
Median [IQR]	30.0 [20.0, 39.0]	25.3 [15.0, 33.0]	25.0 [13.8, 35.2]	27.5 [20.0, 40.0]	38.0 [35.0, 40.0]	36.0 [19.8, 40.0]	28.0 [20.0, 39.0]
**Chest radiation dose (Gy) category**							
No chest radiation	6,607 (68.3%)	1,706 (76.3%)	1,892 (84.6%)	2,728 (79.9%)	22 (8.3%)	49 (62.0%)	13,004 (72.6%)
<10	73 (0.8%)	5 (0.2%)	48 (2.1%)	7 (0.2%)	-	-	133 (0.7%)
10-19	403 (4.2%)	133 (5.9%)	69 (3.1%)	102 (3.0%)	2 (0.8%)	6 (7.6%)	715 (4.0%)
20-29	533 (5.5%)	210 (9.4%)	60 (2.7%)	148 (4.3%)	11 (4.2%)	2 (2.5%)	964 (5.4%)
30-39	542 (5.6%)	85 (3.8%)	82 (3.7%)	92 (2.7%)	90 (34.0%)	5 (6.3%)	896 (5.0%)
≥40	511 (5.3%)	41 (1.8%)	68 (3.0%)	133 (3.9%)	85 (32.1%)	6 (7.6%)	844 (4.7%)
Unknown	1,002 (10.4%)	56 (2.5%)	18 (0.8%)	205 (6.0%)	55 (20.8%)	11 (13.9%)	1,347 (7.5%)
**Chest radiation field**							
No chest radiation	6,607 (68.3%)	1,706 (76.3%)	1,892 (84.6%)	2,728 (79.9%)	22 (8.3%)	49 (62.0%)	13,004 (72.6%)
Axilla	12 (0.1%)	5 (0.2%)	15 (0.7%)	-	2 (0.8%)	-	34 (0.2%)
Mantle	723 (7.5%)	191 (8.5%)	39 (1.7%)	86 (2.5%)	192 (72.5%)	11 (13.9%)	1,242 (6.9%)
Mediastinal	227 (2.3%)	23 (1.0%)	36 (1.6%)	134 (3.9%)	45 (17.0%)	4 (5.1%)	469 (2.6%)
Spine	598 (6.2%)	131 (5.9%)	109 (4.9%)	98 (2.9%)	-	3 (3.8%)	939 (5.2%)
Total body irradiation	223 (2.3%)	67 (3.0%)	69 (3.1%)	10 (0.3%)	-	2 (2.5%)	371 (2.1%)
Whole lung	79 (0.8%)	44 (2.0%)	22 (1.0%)	37 (1.1%)	-	2 (2.5%)	184 (1.0%)
Other	177 (1.8%)	33 (1.5%)	49 (2.2%)	117 (3.4%)	-	1 (1.3%)	377 (2.1%)
Unknown	1,025 (10.6%)	36 (1.6%)	6 (0.3%)	205 (6.0%)	4 (1.5%)	7 (8.9%)	1,283 (7.2%)
**Radiotherapy exposure to the pelvis**							
No	7,191 (74.4%)	1,873 (83.8%)	2,129 (95.2%)	2,287 (67.0%)	179 (67.5%)	68 (86.1%)	13,727 (76.7%)
Yes	1,515 (15.7%)	337 (15.1%)	105 (4.7%)	923 (27.0%)	81 (30.6%)	3 (3.8%)	2,964 (16.6%)
Unknown	965 (10.0%)	26 (1.2%)	3 (0.1%)	205 (6.0%)	5 (1.9%)	8 (10.1%)	1,212 (6.8%)
**Pelvic radiation dose (Gy)**							

Supplementary Table 3 | Demographic and treatment characteristics of female five-year childhood cancer survivors by cohort

Median [IQR]	26.0 [15.0, 36.0]	23.4 [16.8, 36.0]	12.0 [7.5, 38.5]	33.0 [22.0, 43.5]	NA[d]	11.0 [10.5, 11.5]	30.0 [19.0, 39.0]
**Pelvic radiation dose (Gy) category**							
No pelvic radiation	7,191 (74.4%)	1,873 (83.8%)	2,129 (95.2%)	2,287 (67.0%)	179 (67.5%)	68 (86.1%)	13,727 (76.7%)
<10	66 (0.7%)	4 (0.2%)	47 (2.1%)	25 (0.7%)	-	-	142 (0.8%)
10-19	369 (3.8%)	89 (4.0%)	20 (0.9%)	114 (3.3%)	-	2 (2.5%)	594 (3.3%)
20-29	365 (3.8%)	120 (5.4%)	2 (0.1%)	232 (6.8%)	-	-	719 (4.0%)
30-39	398 (4.1%)	66 (3.0%)	6 (0.3%)	216 (6.3%)	81 (30.6%)[e]	-	767 (4.3%)
≥40	295 (3.1%)	57 (2.5%)	25 (1.1%)	336 (9.8%)	-	-	713 (4.0%)
Unknown	987 (10.2%)	27 (1.2%)	8 (0.4%)	205 (6.0%)	5 (1.9%)	9 (11.4%)	1,241 (6.9%)
**Anthracyclines**[f]							
No	4,889 (50.6%)	955 (42.7%)	1,250 (55.9%)	2,095 (61.3%)	155 (58.5%)	36 (45.6%)	9,380 (52.4%)
Yes	3,990 (41.3%)	1,263 (56.5%)	982 (43.9%)	1,201 (35.2%)	98 (37.0%)	36 (45.6%)	7,570 (42.3%)
Unknown	792 (8.2%)	18 (0.8%)	5 (0.2%)	119 (3.5%)	12 (4.5%)	7 (8.9%)	953 (5.3%)
**Doxorubicin**							
No	5,729 (59.2%)	1,377 (61.6%)	1,541 (68.9%)	2,300 (67.4%)	181 (68.3%)	42 (53.2%)	11,170 (62.4%)
Yes	3,150 (32.6%)	841 (37.6%)	691 (30.9%)	996 (29.2%)	84 (31.7%)	30 (38.0%)	5,792 (32.4%)
Unknown	792 (8.2%)	18 (0.8%)	5 (0.2%)	119 (3.5%)	-	7 (8.9%)	941 (5.3%)
**Cumulative doxorubicin dose (mg/m²)**							
Median [IQR]	224.7 [130.4, 358.3]	177.4 [135.1, 256.2]	150.0 [65.0, 300.0]	235.7 [131.7, 346.8]	210.0 [140.0, 280.0]	200.0 [150.0, 300.0]	203.3 [120.0, 340.0]
**Cumulative doxorubicin dose (mg/m²) category**							
0	5,729 (59.2%)	1,377 (61.6%)	1,541 (68.9%)	2,300 (67.4%)	181 (68.3%)	42 (53.2%)	11,170 (62.4%)
<100	502 (5.2%)	121 (5.4%)	188 (8.4%)	95 (2.8%)	5 (1.9%)	1 (1.3%)	912 (5.1%)
100-199	769 (8.0%)	414 (18.5%)	232 (10.4%)	347 (10.2%)	20 (7.5%)	13 (16.5%)	1,795 (10.0%)
200-299	590 (6.1%)	124 (5.5%)	62 (2.8%)	207 (6.1%)	38 (14.3%)	5 (6.3%)	1,026 (5.7%)
300-399	568 (5.9%)	146 (6.5%)	77 (3.4%)	203 (5.9%)	11 (4.2%)	7 (8.9%)	1,012 (5.7%)
≥400	474 (4.9%)	35 (1.6%)	124 (5.5%)	137 (4.0%)	6 (2.3%)	3 (3.8%)	779 (4.4%)
Unknown	1,039 (10.7%)	19 (0.8%)	13 (0.6%)	126 (3.7%)	4 (1.5%)	8 (10.1%)	1,209 (6.8%)
**Daunorubicin**							
No	7,660 (79.2%)	1,618 (72.4%)	1,795 (80.2%)	3,239 (94.8%)	253 (95.5%)	65 (82.3%)	14,630 (81.7%)
Yes	1,219 (12.6%)	600 (26.8%)	437 (19.5%)	57 (1.7%)	-	7 (8.9%)	2,320 (13.0%)
Unknown	792 (8.2%)	18 (0.8%)	5 (0.2%)	119 (3.5%)	12 (4.5%)	7 (8.9%)	953 (5.3%)
**Cumulative daunorubicin (mg/m²)**							
Median [IQR]	151.0 [100.0, 319.4]	87.5 [50.0, 106.7]	120.0 [120.0, 175.0]	255.7 [140.8, 419.7]	-	150.0 [120.0, 247.5]	120.0 [98.1, 234.1]
**Cumulative daunorubicin dose (mg/m²) category**							
0	7,660 (79.2%)	1,618 (72.4%)	1,795 (80.2%)	3,239 (94.8%)	253 (95.5%)	65 (82.3%)	14,630 (81.7%)
<100	263 (2.7%)	339 (15.2%)	16 (0.7%)	5 (0.1%)	-	-	623 (3.5%)
100-199	373 (3.9%)	198 (8.9%)	361 (16.1%)	17 (0.5%)	-	4 (5.1%)	953 (5.3%)
≥200	494 (5.1%)	62 (2.8%)	51 (2.3%)	35 (1.0%)	-	3 (3.8%)	645 (3.6%)
Unknown	881 (9.1%)	19 (0.8%)	14 (0.6%)	119 (3.5%)	12 (4.5%)	7 (8.9%)	1,052 (5.9%)
**Epirubicin**							
No	8,877 (91.8%)	2,217 (99.2%)	2,104 (94.1%)	3,116 (91.2%)	251 (94.7%)	72 (91.1%)	16,637 (92.9%)
Yes	2 (0.0%)	1 (0.0%)	128 (5.7%)	180 (5.3%)	14 (5.3%)	-	325 (1.8%)

Supplementary Table 3 | Demographic and treatment characteristics of female five-year childhood cancer survivors by cohort

Unknown	792 (8.2%)	18 (0.8%)	5 (0.2%)	119 (3.5%)	-	7 (8.9%)	941 (5.3%)
**Idarubicin**							
No	8,814 (91.1%)	2,198 (98.3%)	2,212 (98.9%)	3,296 (96.5%)	253 (95.5%)	70 (88.6%)	16,843 (94.1%)
Yes	65 (0.7%)	20 (0.9%)	20 (0.9%)	-	-	2 (2.5%)	107 (0.6%)
Unknown	792 (8.2%)	18 (0.8%)	5 (0.2%)	119 (3.5%)	12 (4.5%)	7 (8.9%)	953 (5.3%)
**Alkylating agents**							
No	4,003 (41.4%)	947 (42.4%)	1,152 (51.5%)	1,597 (46.8%)	100 (37.7%)	33 (41.8%)	7,832 (43.7%)
Yes	4,876 (50.4%)	1,271 (56.8%)	1,080 (48.3%)	1,699 (49.8%)	153 (57.7%)	39 (49.4%)	9,118 (50.9%)
Unknown	792 (8.2%)	18 (0.8%)	5 (0.2%)	119 (3.5%)	12 (4.5%)	7 (8.9%)	953 (5.3%)
**CED[g] dose (mg/m²)**							
0	4,093 (42.3%)	956 (42.8%)	1,160 (51.9%)	1,608 (47.1%)	100 (37.7%)	34 (43.0%)	7,951 (44.4%)
<6000	1,687 (17.4%)	489 (21.9%)	265 (11.8%)	606 (17.7%)	-	22 (27.8%)	3,069 (17.1%)
6000-17999	1,876 (19.4%)	631 (28.2%)	563 (25.2%)	819 (24.0%)	-	10 (12.7%)	3,899 (21.8%)
≥18000	561 (5.8%)	139 (6.2%)	192 (8.6%)	222 (6.5%)	-	3 (3.8%)	1,117 (6.2%)
Unknown	1,454 (15.0%)	21 (0.9%)	57 (2.5%)	160 (4.7%)	165 (62.3%)	10 (12.7%)	1,867 (10.4%)
**Epipodophyllotoxins**							
No	7,567 (78.2%)	1,402 (62.7%)	1,796 (80.3%)	2,531 (74.1%)	83 (31.3%)	55 (69.6%)	13,434 (75.0%)
Yes	1,312 (13.6%)	816 (36.5%)	436 (19.5%)	765 (22.4%)	-	17 (21.5%)	3,346 (18.7%)
Unknown	792 (8.2%)	18 (0.8%)	5 (0.2%)	119 (3.5%)	182 (68.7%)	7 (8.9%)	1,123 (6.3%)
**Vinca alkaloids**							
No	3,351 (34.7%)	706 (31.6%)	653 (29.2%)	1,367 (40.0%)	83 (31.3%)	34 (43.0%)	6,194 (34.6%)
Yes	5,528 (57.2%)	1,512 (67.6%)	1,579 (70.6%)	1,929 (56.5%)	-	38 (48.1%)	10,586 (59.1%)
Unknown	792 (8.2%)	18 (0.8%)	5 (0.2%)	119 (3.5%)	182 (68.7%)	7 (8.9%)	1,123 (6.3%)
**Platinum compounds**							
No	7,817 (80.8%)	1,870 (83.6%)	1,911 (85.4%)	2,473 (72.4%)	83 (31.3%)	65 (82.3%)	14,219 (79.4%)
Yes	1,062 (11.0%)	348 (15.6%)	321 (14.3%)	823 (24.1%)	-	7 (8.9%)	2,561 (14.3%)
Unknown	792 (8.2%)	18 (0.8%)	5 (0.2%)	119 (3.5%)	182 (68.7%)	7 (8.9%)	1,123 (6.3%)
**Antimetabolites**							
No	5,012 (51.8%)	1,151 (51.5%)	1,261 (56.4%)	2,816 (82.5%)	83 (31.3%)	53 (67.1%)	10,376 (58.0%)
Yes	3,867 (40.0%)	1,067 (47.7%)	971 (43.4%)	480 (14.1%)	-	19 (24.1%)	6,404 (35.8%)
Unknown	792 (8.2%)	18 (0.8%)	5 (0.2%)	119 (3.5%)	182 (68.7%)	7 (8.9%)	1,123 (6.3%)

CCSS = Childhood Cancer Survivor Study; CED = Cyclophosphamide Equivalent Dose; DCCSS-LATER = Dutch Childhood Cancer Survivor Study LATER; DCIS = Ductal carcinoma in situ; DHL = Dutch Hodgkin Late Effects cohort; FCCSS = French Childhood Cancer Survivor Study; Gy = Gray; IQR = Interquartile range; NA = Not applicable; SBC = Subsequent breast cancer; SCCSS = Swiss Childhood Cancer Survivor Study; SJLIFE = St. Jude Lifetime Cohort Study; yr = year

[a]Included the ICCC-3 classification groups "Hepatic Tumor", "Other and Unspecified", and "Unclassified".

[b]Follow-up time was calculated from five years after a primary cancer diagnosis to the date of subsequent breast cancer diagnosis, death, or the date of the last follow-up observation, whichever occurred first.

[c]Included both invasive and DCIS breast cancer.

[d]Precise pelvic radiation dose information was not available in the DHL.

[e]Dose of pelvic radiation information was not available for the DHL. We assume the survivors in the DHL who had pelvic radiotherapy received 30 Gy radiotherapy exposure to the pelvis since Hodgkin lymphoma patients usually receive 30 Gy pelvic radiation.

[f]Anthracyclines included doxorubicin, daunorubicin, epirubicin, and idarubicin.

[g]Cyclophosphamide Equivalent Dose calculation: CED (mg/m^2) = 1.0 (cumulative cyclophosphamide dose (mg/m^2)) + 0.244 (cumulative ifosfamide dose (mg/m^2)) + 0.857 (cumulative procarbazine dose (mg/m^2)) + 14.286 (cumulative chlorambucil dose (mg/m^2)) + 15.0 (cumulative BCNU (carmustine) dose (mg/m^2)) + 16.0 (cumulative CCNU (lomustine) dose (mg/m^2)) + 40 (cumulative melphalan dose (mg/m^2)) + 50 (cumulative Thio-TEPA (thiotepa) dose (mg/m^2)) + 100 (cumulative nitrogen mustard dose (mg/m^2)) + 8.823 (cumulative busulfan dose (mg/m^2)).

Supplementary Table 4 | Sensitivity analyses for risk of subsequent breast cancer by excluding each cohort on a one-by-one basis

	Without CCSS[a]				Without SJLIFE[b]				Without DCCSS-LATER[c]				Without FCCSS[d]				Without DHL[e]				Without SCCSS[f]			
	No. SBC	Total	HR	95%CI	No. SBC	Total	HR	95%CI	No. SBC	Total	HR	95%CI	No. SBC	Total	HR	95%CI	No. SBC	Total	HR	95%CI	No. SBC	Total	HR	95%CI
**Cumulative doxorubicin dose, mg/m²**																								
0	179 (54.2%)	5,441 (66.1%)	1.0	Ref.	395 (56.0%)	9,793 (62.5%)	1.0	Ref.	409 (55.2%)	9,629 (61.5%)	1.0	Ref.	370 (56.5%)	8,870 (61.2%)	1.0	Ref.	379 (52.9%)	10,989 (62.3%)	1.0	Ref.	423 (55.5%)	11,128 (62.4%)	1.0	Ref.
<100	6 (1.8%)	410 (5.0%)	1.18	0.47-2.97	15 (2.1%)	791 (5.0%)	2.10	1.01-4.35	14 (1.9%)	724 (4.6%)	1.90	0.90-3.99	13 (2.0%)	817 (5.6%)	1.77	0.80-3.93	16 (2.2%)	907 (5.1%)	1.75	0.88-3.49	16 (2.1%)	911 (5.1%)	1.74	0.88-3.47
100-199	42 (12.7%)	1,026 (12.5%)	2.34	1.49-3.67	54 (7.7%)	1,381 (8.8%)	1.72	1.20-2.47	66 (8.9%)	1,563 (10.0%)	1.84	1.34-2.54	52 (7.9%)	1,448 (10.0%)	1.63	1.15-2.30	66 (9.2%)	1,775 (10.1%)	1.75	1.28-2.39	65 (8.5%)	1,782 (10.0%)	1.66	1.21-2.26
200-299	27 (8.2%)	436 (5.3%)	2.35	1.39-3.99	61 (8.7%)	902 (5.8%)	2.74	1.99-3.78	66 (8.9%)	964 (6.2%)	2.59	1.90-3.53	55 (8.4%)	819 (5.7%)	2.41	1.73-3.36	60 (8.4%)	988 (5.6%)	2.46	1.81-3.34	66 (8.7%)	1,021 (5.7%)	2.42	1.78-3.28
300-399	28 (8.5%)	444 (5.4%)	3.03	1.80-5.11	52 (7.4%)	866 (5.5%)	2.02	1.40-2.92	60 (8.1%)	935 (6.0%)	2.33	1.66-3.26	55 (8.4%)	809 (5.6%)	2.42	1.71-3.43	62 (8.6%)	1,001 (5.7%)	2.31	1.67-3.19	63 (8.3%)	1,005 (5.6%)	2.30	1.67-3.19
≥400	23 (7.0%)	305 (3.7%)	4.09	2.35-7.14	51 (7.2%)	744 (4.7%)	2.42	1.68-3.48	49 (6.6%)	655 (4.2%)	2.72	1.91-3.88	53 (8.1%)	642 (4.4%)	2.84	1.99-4.06	57 (7.9%)	773 (4.4%)	2.74	1.96-3.82	57 (7.5%)	776 (4.4%)	2.78	1.99-3.87
Unknown	25 (7.6%)	170 (2.1%)	-	-	77 (10.9%)	1,190 (7.6%)	-	-	77 (10.4%)	1,196 (7.6%)	-	-	57 (8.7%)	1,083 (7.5%)	-	-	77 (10.7%)	1,205 (6.8%)	-	-	72 (9.4%)	1,201 (6.7%)	-	-
**Cumulative daunorubicin dose, mg/m²**																								
0	284 (86.1%)	6,970 (84.7%)	1.0	Ref.	619 (87.8%)	13,012 (83.1%)	1.0	Ref.	648 (87.4%)	12,835 (81.9%)	1.0	Ref.	581 (88.7%)	11,391 (78.6%)	1.0	Ref.	619 (86.3%)	14,377 (81.5%)	1.0	Ref.	669 (87.8%)	14,565 (81.7%)	1.0	Ref.
<100	5 (1.5%)	360 (4.4%)	1.42	0.56-3.61	2 (0.3%)	284 (1.8%)	0.48	0.12-1.93	7 (0.9%)	607 (3.9%)	1.00	0.47-2.16	7 (1.1%)	618 (4.3%)	1.01	0.47-2.18	7 (1.0%)	623 (3.5%)	0.98	0.46-2.09	7 (0.9%)	623 (3.5%)	0.97	0.45-2.07
100-199	11 (3.3%)	580 (7.0%)	1.60	0.81-3.19	11 (1.6%)	755 (4.8%)	0.72	0.34-1.55	11 (1.5%)	592 (3.8%)	0.81	0.41-1.61	15 (2.3%)	936 (6.5%)	1.06	0.59-1.92	16 (2.2%)	953 (5.4%)	0.98	0.55-1.75	16 (2.1%)	949 (5.3%)	0.98	0.55-1.75
≥200	6 (1.8%)	151 (1.8%)	2.99	1.14-7.85	15 (2.1%)	583 (3.7%)	1.07	0.58-1.98	17 (2.3%)	594 (3.8%)	1.29	0.72-2.32	14 (2.1%)	610 (4.2%)	0.99	0.52-1.89	17 (2.4%)	645 (3.7%)	1.21	0.68-2.15	16 (2.1%)	642 (3.6%)	1.21	0.68-2.14
Unknown	24 (7.3%)	171 (2.1%)	-	-	58 (8.2%)	1,033 (6.6%)	-	-	58 (7.8%)	1,038 (6.6%)	-	-	38 (5.8%)	933 (6.4%)	-	-	58 (8.1%)	1,040 (5.9%)	-	-	54 (7.1%)	1,045 (5.9%)	-	-
**Epirubicin**																								
No	297 (90.0%)	7,760 (94.3%)	1.0	Ref.	640 (90.8%)	14,420 (92.0%)	1.0	Ref.	677 (91.4%)	14,533 (92.8%)	1.0	Ref.	616 (94.0%)	13,521 (93.3%)	1.0	Ref.	654 (91.2%)	16,386 (92.9%)	1.0	Ref.	701 (92.0%)	16,565 (92.9%)	1.0	Ref.
Yes	9 (2.7%)	323 (3.9%)	3.64	1.72-7.72	9 (1.3%)	324 (2.1%)	3.35	1.63-6.89	8 (1.1%)	197 (1.3%)	4.13	1.97-8.66	3 (0.5%)	145 (1.0%)	3.99	1.31-12.11	7 (1.0%)	311 (1.8%)	2.61	1.11-6.13	9 (1.2%)	325 (1.8%)	3.36	1.65-6.86
Unknown	24 (7.3%)	149 (1.8%)	-	-	56 (7.9%)	923 (5.9%)	-	-	56 (7.6%)	936 (6.0%)	-	-	36 (5.5%)	822 (5.7%)	-	-	56 (7.8%)	941 (5.3%)	-	-	52 (6.8%)	934 (5.2%)	-	-
**Chest radiotherapy field and dose**																								
No chest radiotherapy	96 (29.1%)	639,7 (77.7%)	1.0	Ref.	229 (32.5%)	11,298 (72.1%)	1.0	Ref.	226 (30.5%)	11,112 (70.9%)	1.0	Ref.	205 (31.3%)	10,276 (70.9%)	1.0	Ref.	250 (34.9%)	12,982 (73.6%)	1.0	Ref.	244 (32.0%)	12,955 (72.7%)	1.0	Ref.
High-dose mantle (≥36 Gy)	89 (27.0%)	237 (2.9%)	11.20	7.10-17.67	223 (31.6%)	652 (4.2%)	8.55	6.55-11.16	233 (31.4%)	669 (4.3%)	9.33	7.20-12.11	222 (33.9%)	651 (4.5%)	8.67	6.69-11.24	192 (26.8%)	591 (3.4%)	9.03	7.01-11.64	231 (30.3%)	690 (3.9%)	8.37	6.56-10.69
Low-dose mantle(<36 Gy)	43 (13.0%)	252 (3.1%)	5.83	3.63-9.37	69 (9.8%)	377 (2.4%)	4.29	3.02-6.11	90 (12.1%)	506 (3.2%)	4.70	3.43-6.44	89 (13.6%)	485 (3.3%)	4.66	3.40-6.38	81 (11.3%)	477 (2.7%)	4.85	3.54-6.65	93 (12.2%)	523 (2.9%)	4.61	3.40-6.25
Mediastinal	18 (5.5%)	242 (2.9%)	1.40	0.59-3.28	31 (4.4%)	446 (2.8%)	1.59	0.98-2.60	33 (4.5%)	433 (2.8%)	1.83	1.13-2.97	23 (3.5%)	335 (2.3%)	1.45	0.83-2.55	27 (3.8%)	424 (2.4%)	1.64	1.00-2.69	33 (4.3%)	465 (2.6%)	1.73	1.09-2.75
TBI	11 (3.3%)	148 (1.8%)	9.42	4.44-19.99	16 (2.3%)	304 (1.9%)	5.47	2.92-10.25	19 (2.6%)	302 (1.9%)	7.31	4.08-13.10	21 (3.2%)	361 (2.5%)	7.55	4.30-13.26	22 (3.1%)	371 (2.1%)	7.38	4.30-12.69	21 (2.8%)	369 (2.1%)	6.93	4.04-11.86
Whole lung	14 (4.2%)	105 (1.3%)	8.33	4.24-16.38	20 (2.8%)	140 (0.9%)	7.29	4.36-12.19	19 (2.6%)	162 (1.0%)	6.83	4.02-11.60	18 (2.7%)	147 (1.0%)	8.42	4.86-14.59	23 (3.2%)	184 (1.0%)	7.74	4.77-12.56	21 (2.8%)	182 (1.0%)	7.04	4.32-11.46
Other	39 (11.8%)	541 (6.6%)	4.58	2.97-7.07	59 (8.4%)	1,152 (7.4%)	2.54	1.79-3.60	62 (8.4%)	1,158 (7.4%)	2.95	2.09-4.16	30 (4.6%)	1,101 (7.6%)	1.42	0.88-2.29	63 (8.8%)	1,316 (7.5%)	2.67	1.91-3.73	62 (8.1%)	1,312 (7.4%)	2.51	1.80-3.51
Unknown	20 (6.1%)	310 (3.8%)	-	-	58 (8.2%)	1,298 (8.3%)	-	-	59 (8.0%)	1,324 (8.5%)	-	-	47 (7.2%)	1,132 (7.8%)	-	-	59 (8.2%)	1,293 (7.3%)	-	-	57 (7.5%)	1,328 (7.5%)	-	-
**Pelvic radiotherapy ≥5 Gy**																								
No	192 (58.2%)	6,551 (79.6%)	1.0	Ref.	452 (64.1%)	1,1876 (75.8%)	1.0	Ref.	469 (63.3%)	11,620 (74.2%)	1.0	Ref.	462 (70.5%)	11,453 (79.1%)	1.0	Ref.	462 (64.4%)	13,572 (76.9%)	1.0	Ref.	488 (64.0%)	13,683 (76.8%)	1.0	Ref.
Yes	120 (36.4%)	1,427 (17.3%)	1.36	1.00-1.86	201 (28.5%)	2,577 (16.4%)	1.03	0.83-1.28	217 (29.3%)	2,813 (18.0%)	0.94	0.76-1.15	151 (23.1%)	1,999 (13.8%)	0.83	0.65-1.06	200 (27.9%)	2,830 (16.0%)	0.90	0.73-1.11	221 (29.0%)	2,909 (16.3%)	0.97	0.79-1.19
Unknown	18 (5.5%)	254 (3.1%)	-	-	52 (7.4%)	1,214 (7.7%)	-	-	55 (7.4%)	1,233 (7.9%)	-	-	42 (6.4%)	1,036 (7.2%)	-	-	55 (7.7%)	1,236 (7.0%)	-	-	53 (7.0%)	1,232 (6.9%)	-	-

Supplementary Table 4 | Sensitivity analyses for risk of subsequent breast cancer by excluding each cohort on a one-by-one basis

	Without CCSS[a]				Without SJLIFE[b]				Without DCCSS-LATER[c]				Without FCCSS[d]				Without DHL[e]				Without SCCSS[f]			
	**No. SBC**	**Total**	**HR**	**95%CI**	**No. SBC**	**Total**	**HR**	**95%CI**	**No. SBC**	**Total**	**HR**	**95%CI**	**No. SBC**	**Total**	**HR**	**95%CI**	**No. SBC**	**Total**	**HR**	**95%CI**	**No. SBC**	**Total**	**HR**	**95%CI**
**Age at primary childhood cancer diagnosis, yr**																								
<5	46 (13.9%)	3,710 (45.1%)	1.0	Ref.	59 (8.4%)	6,403 (40.9%)	1.0	Ref.	58 (7.8%)	6,327 (40.4%)	1.0	Ref.	36 (5.5%)	5,705 (39.4%)	1.0	Ref.	66 (9.2%)	7,376 (41.8%)	1.0	Ref.	65 (8.5%)	7,359 (41.3%)	1.0	Ref.
5-9	33 (10.0%)	1,761 (21.4%)	0.90	0.54-1.52	59 (8.4%)	3,320 (21.2%)	1.14	0.75-1.74	58 (7.8%)	3,219 (20.5%)	1.25	0.81-1.92	46 (7.0%)	3,081 (21.3%)	1.20	0.72-2.02	65 (9.1%)	3,781 (21.4%)	1.13	0.76-1.69	64 (8.4%)	3,778 (21.2%)	1.13	0.76-1.69
10-14	117 (35.5%)	1,726 (21.0%)	1.87	1.23-2.83	240 (34.0%)	3,458 (22.1%)	1.92	1.37-2.69	257 (34.7%)	3,459 (22.1%)	2.25	1.60-3.17	220 (33.6%)	3,186 (22.0%)	2.22	1.46-3.36	265 (37.0%)	3,909 (22.2%)	2.01	1.46-2.76	266 (34.9%)	3,912 (21.9%)	2.03	1.47-2.79
15-21	134 (40.6%)	1,035 (12.6%)	1.51	0.93-2.45	347 (49.2%)	2,486 (15.9%)	1.81	1.27-2.58	368 (49.7%)	2,661 (17.0%)	1.97	1.38-2.81	353 (53.9%)	2,516 (17.4%)	1.89	1.24-2.88	321 (44.8%)	2,572 (14.6%)	1.86	1.33-2.59	367 (48.2%)	2,775 (15.6%)	1.91	1.37-2.66
**CED[g], mg/m²**																								
None	120 (36.4%)	3,858 (46.9%)	1.0	Ref.	279 (39.6%)	6,995 (44.6%)	1.0	Ref.	285 (38.5%)	6,791 (43.3%)	1.0	Ref.	259 (39.5%)	6,343 (43.8%)	1.0	Ref.	266 (37.1%)	7,851 (44.5%)	1.0	Ref.	296 (38.8%)	7,917 (44.4%)	1.0	Ref.
<6000	48 (14.5%)	1,382 (16.8%)	1.03	0.68-1.57	76 (10.8%)	2,580 (16.5%)	0.87	0.65-1.17	91 (12.3%)	2,804 (17.9%)	0.86	0.66-1.13	71 (10.8%)	2,463 (17.0%)	0.84	0.62-1.14	94 (13.1%)	3,069 (17.4%)	0.87	0.67-1.14	90 (11.8%)	3,047 (17.1%)	0.85	0.65-1.11
6000-17999	80 (24.2%)	2,023 (24.6%)	0.85	0.57-1.26	159 (22.6%)	3,268 (20.9%)	1.10	0.87-1.39	180 (24.3%)	3,336 (21.3%)	1.00	0.80-1.26	160 (24.4%)	3,080 (21.3%)	1.06	0.84-1.34	192 (26.8%)	3,899 (22.1%)	1.03	0.82-1.28	189 (24.8%)	3,889 (21.8%)	1.01	0.81-1.26
≥18000	23 (7.0%)	556 (6.8%)	0.78	0.43-1.42	43 (6.1%)	978 (6.2%)	1.47	0.99-2.18	38 (5.1%)	925 (5.9%)	1.18	0.79-1.75	38 (5.8%)	895 (6.2%)	1.26	0.84-1.89	47 (6.6%)	1,117 (6.3%)	1.21	0.84-1.76	46 (6.0%)	1,114 (6.3%)	1.16	0.80-1.68
Unknown	59 (17.9%)	413 (5.0%)	-	-	148 (21.0%)	1,846 (11.8%)	-	-	147 (19.8%)	1,810 (11.6%)	-	-	127 (19.4%)	1,707 (11.8%)	-	-	118 (16.5%)	1,702 (9.6%)	-	-	141 (18.5%)	1,857 (10.4%)	-	-

CCSS = Childhood Cancer Survivor Study; CED = Cyclophosphamide Equivalent Dose; CI = Confidence interval; DCCSS-LATER = Dutch Childhood Cancer Survivor Study LATER; DHL = Dutch Hodgkin Late Effects cohort; FCCSS = French Childhood Cancer Survivor Study; Gy = Gray; HR = Hazard ratio; NA = Not applicable; No. = number; SBC = Subsequent breast cancer; SCCSS = Swiss Childhood Cancer Survivor Study; SJLIFE = St. Jude Lifetime Cohort Study; TBI = Total Body Irradiation; yr = year

[a]In total, 9,671 survivors with 452 SBC cases were excluded.

[b]In total, 2,236 survivors with 77 SBC cases were excluded.

[c]In total, 2,237 survivors with 41 SBC cases were excluded.

[d]In total, 3,415 survivors with 127 SBC cases were excluded.

[e]In total, 265 survivors with 65 SBC cases were excluded.

[f]In total, 79 survivors with 20 SBC cases were excluded.

[g]Cyclophosphamide Equivalent Dose calculation: CED (mg/m^2) = 1.0 (cumulative cyclophosphamide dose (mg/m^2)) + 0.244 (cumulative ifosfamide dose (mg/m^2)) + 0.857 (cumulative procarbazine dose (mg/m^2)) + 14.286 (cumulative chlorambucil dose (mg/m^2)) + 15.0 (cumulative BCNU (carmustine) dose (mg/m^2)) + 16.0 (cumulative CCNU (lomustine) dose (mg/m^2)) + 40 (cumulative melphalan dose (mg/m^2)) + 50 (cumulative Thio-TEPA (thiotepa) dose (mg/m^2)) + 100 (cumulative nitrogen mustard dose (mg/m^2)) + 8.823 (cumulative busulfan dose (mg/m^2)).

Supplementary Table 5 | Multivariable Cox proportional hazard regression analyses for subsequent breast cancer in female five-year childhood cancer survivors in each cohort[a]

	CCSS[b]		SJLIFE[b]		DCCSS-LATER[c]		FCCSS[d]		DHL[e]	
	**HR**	**95% CI**	**HR**	**95% CI**	**HR**	**95% CI**	**HR**	**95% CI**	**HR**	**95% CI**
**Cumulative doxorubicin dose, mg/m²**										
0	1.0	Ref.	1.0	Ref.	1.0	Ref.	1.0	Ref.	1.0	Ref.
<100	2.58	0.99-6.74	0.99	0.13-7.70	1.00	0.19-5.11	1.40	0.42-4.70	1.49	0.45-4.91
100-199	1.34	0.85-2.10	2.49	1.10-5.65	1.04	0.27-4.02	2.23	1.12-4.43		
200-299	2.60	1.80-3.75	1.42	0.54-3.74	0.97	0.13-7.55	2.79	1.39-5.59	1.74	0.76-3.96
300-399	1.96	1.30-2.97	4.82	2.16-10.75	3.51	1.02-12.10	1.60	0.68-3.74	1.50	0.35-6.34
≥400	2.22	1.46-3.39	6.73	2.54-17.83	5.49	1.97-15.25	2.42	0.90-6.49	1.84	0.24-14.00
**Cumulative daunorubicin dose, mg/m²**										
0	1.0	Ref.	1.0	Ref.						
<100	0.47	0.11-1.89	2.28	0.83-6.27						
100-199	0.47	0.16-1.40	3.00	1.05-8.60						
≥200	0.83	0.40-1.70	4.06	0.82-20.17						
**Daunorubicin**										
No					1.0	Ref.	1.0	Ref.		
Yes					1.53	0.49-4.79	1.92	0.57-6.38		
**Epirubicin**										
No					1.0	Ref.	1.0	Ref.		
Yes					1.80	0.24-13.79	3.18	1.12-9.05		
**Chest radiotherapy field and dose**										
No chest radiotherapy	1.0	Ref.	1.0	Ref.			1.0	Ref.		
High-dose mantle (≥36 Gy)	7.65	5.76-10.16	11.51	5.12-25.84			9.05	4.54-18.07		
Low-dose mantle (<36 Gy)	4.04	2.72-6.00	8.40	3.95-17.87			2.06	0.47-9.14		
Mediastinal	1.66	0.94-2.95	1.86	0.24-14.52			1.84	0.75-4.51		
TBI	6.75	3.25-14.02	12.43	3.76-41.08			5.41	0.67-43.44		
Whole lung	6.32	3.05-13.11	11.83	3.07-45.57			3.72	1.28-10.83		
Other	1.37	0.78-2.40	2.65	0.85-8.23			7.43	4.53-12.17		
**Chest radiotherapy fields**										
No chest radiotherapy					1.0	Ref.				
Mantle					8.11	2.99-21.95				
TBI & Whole lung					15.43	4.65-51.27				
Other fields					0.67	0.20-2.30				
**Chest radiotherapy fields**										
Non-mantle									1.0	Ref.
Mantle									2.80	1.18-6.63
**Pelvic radiotherapy ≥5 Gy**										
No	1.0	Ref.	1.0	Ref.	1.0	Ref.	1.0	Ref.	1.0	Ref.
Yes	0.79	0.59-1.06	0.83	0.45-1.50	0.65	0.17-2.53	2.18	1.38-3.45	1.49	0.87-2.53
**Age at primary childhood cancer diagnosis, yr**										
<5	1.0	Ref.	1.0	Ref.	1.0	Ref.	1.0	Ref.	1.0	Ref.
5-9	1.62	0.83-3.18	0.97	0.27-3.45	0.48	0.16-1.45	1.10	0.57-2.11		
10-14	2.61	1.49-4.57	3.30	1.19-9.16	0.93	0.36-2.41	1.86	1.06-3.28		
15-21	2.50	1.43-4.38	2.08	0.71-6.05	1.36	0.47-3.92	2.17	1.09-4.30	0.64	0.30-1.36
**CED[f], mg/m²**										
None	1.0	Ref.	1.0	Ref.	1.0	Ref.	1.0	Ref.		

Supplementary Table 5 | Multivariable Cox proportional hazard regression analyses for subsequent breast cancer in female five-year childhood cancer survivors in each cohort[a]

	CCSS[b]		SJLIFE[b]		DCCSS-LATER[c]		FCCSS[d]		DHL[e]	
	**HR**	**95% CI**	**HR**	**95% CI**	**HR**	**95% CI**	**HR**	**95% CI**	**HR**	**95% CI**
<6000	0.78	0.55-1.11	0.96	0.45-2.08	0.73	0.18-2.99	1.04	0.57-1.90		
6000-17999	1.11	0.86-1.45	0.74	0.40-1.36	1.40	0.57-3.42	0.93	0.54-1.62		
≥18000	1.61	1.00-2.59	0.43	0.14-1.38	1.49	0.51-4.31	1.05	0.46-2.39		

CCSS = Childhood Cancer Survivor Study; CED = Cyclophosphamide Equivalent Dose; CI = Confidence interval; DCCSS-LATER = Dutch Childhood Cancer Survivor Study LATER; DHL = Dutch Hodgkin Late Effects cohort; FCCSS = French Childhood Cancer Survivor Study; Gy = Gray; HR = Hazard ratio; SBC = Subsequent breast cancer; SJLIFE = St. Jude Lifetime Cohort Study; TBI = Total Body Irradiation; yr = year

[a]The results of the multivariable Cox proportional hazard regression analyses in the Swiss Childhood Cancer Survivor Study (SCCSS) are not provided due to low numbers in the case-cohort data.

[b]The model included cumulative doxorubicin and daunorubicin dose (categorical variables), chest radiotherapy field and dose (categorical variable), pelvic radiotherapy ≥5 Gy (yes/no), age at primary childhood cancer diagnosis (categorical variable), and CED (categorical variable).

[c]The model included cumulative doxorubicin dose (categorical variable), daunorubicin (yes/no), epirubicin (yes/no), chest radiotherapy fields (categorical variable), pelvic radiotherapy ≥5 Gy (yes/no), age at primary childhood cancer diagnosis (categorical variable), and CED (categorical variable).

[d]The model included cumulative doxorubicin dose (categorical variable), daunorubicin (yes/no), epirubicin (yes/no), chest radiotherapy field and dose (categorical variable), pelvic radiotherapy ≥5 Gy (yes/no), age at primary childhood cancer diagnosis (categorical variable), and CED (categorical variable).

[e]The model included cumulative doxorubicin dose (categorical variable), chest radiotherapy fields (categorical variable), pelvic radiotherapy ≥5 Gy (yes/no), and age at primary childhood cancer diagnosis (categorical variable).

[f]Cyclophosphamide Equivalent Dose calculation: CED (mg/m^2) = 1.0 (cumulative cyclophosphamide dose (mg/m^2)) + 0.244 (cumulative ifosfamide dose (mg/m^2)) + 0.857 (cumulative procarbazine dose (mg/m^2)) + 14.286 (cumulative chlorambucil dose (mg/m^2)) + 15.0 (cumulative BCNU (carmustine) dose (mg/m^2)) + 16.0 (cumulative CCNU (lomustine) dose (mg/m^2)) + 40 (cumulative melphalan dose (mg/m^2)) + 50 (cumulative Thio-TEPA (thiotepa) dose (mg/m^2)) + 100 (cumulative nitrogen mustard dose (mg/m^2)) + 8.823 (cumulative busulfan dose (mg/m^2)).

Supplementary Table 6 | Subsequent breast cancer ascertainment/validation process for each participating study

	Subsequent breast cancer	
**Studies**	**Ascertainment**	**Validation**
CCSS	Record linkage to the National death index;<br><br>Initial self- or proxy-reports	Medical records including pathology reports
SJLIFE	Record linkage to the Cancer registry follow-up and the National Death Index;<br><br>Prospective follow-up at St. Jude with breast imaging, self-report or next of kin reported	Medical records including pathology reports
DCCSS-LATER	Medical records;<br><br>Record linkage to the Population-based Netherlands Cancer Registry and the Nationwide network and registry of histo- and cytopathology in the Netherlands (PALGA [Dutch Pathology Registry])	Pathology reports
FCCSS	Hospital clinical files and Long term follow-up visits;<br><br>Record linkage to the National death certificate data and the National Public and Private Hospital and National Health Insurance Database;<br><br>Self-completed questionnaire	Pathology reports
SCCSS	Medical records including pathology reports;<br><br>Record linkage to the Cantonal cancer registries and the Cause-of death statistics;<br><br>Self-report	Medical records including pathology reports
DHL	Medical records and Questionnaires sent to general practitioners;<br><br>Record linkage to the Population-based Netherlands Cancer Registry	Pathology reports

CCSS = Childhood Cancer Survivor Study; system; DCCSS-LATER = Dutch Childhood Cancer Survivor Study LATER; DHL = Dutch Hodgkin Late Effects cohort; FCCSS = French Childhood Cancer Survivor Study; SCCSS = Swiss Childhood Cancer Survivor Study; SJLIFE = St. Jude Lifetime Cohort Study

Supplementary Table 7 | Selection procedures for chemotherapeutic agents other than anthracyclines and alkylating agents in multivariable Cox proportional hazard regression analyses for subsequent breast cancer risk among female five-year childhood cancer survivors[a]

			Base model		Base model + Epipodophyllotoxins		Base model + Vinca alkaloids		Base model + Platinum compounds		Base model + Antimetabolites	
	No. SBC (n)[b]	Total (n)	HR	95% CI	HR	95% CI	HR	95% CI	HR	95% CI	HR	95% CI
**Cumulative doxorubicin dose, mg/m²**												
0	431 (55.1%)	11,170 (62.4%)	1.0	Ref.	1.0	Ref.	1.0	Ref.	1.0	Ref.	1.0	Ref.
<100	16 (2.0%)	912 (5.1%)	1.76	0.88-3.51	1.71	0.86-3.41	1.79	0.90-3.58	1.75	0.88-3.49	1.77	0.89-3.52
100-199	69 (8.8%)	1,795 (10.0%)	1.77	1.30-2.42	1.73	1.27-2.36	1.82	1.33-2.49	1.76	1.29-2.40	1.78	1.31-2.42
200-299	67 (8.6%)	1,026 (5.7%)	2.50	1.85-3.40	2.43	1.79-3.31	2.59	1.90-3.54	2.49	1.84-3.37	2.51	1.85-3.41
300-399	64 (8.2%)	1,012 (5.7%)	2.33	1.68-3.23	2.21	1.59-3.08	2.39	1.72-3.34	2.32	1.67-3.23	2.36	1.69-3.29
≥400	58 (7.4%)	779 (4.4%)	2.78	1.99-3.88	2.73	1.96-3.82	2.83	2.02-3.96	2.78	1.99-3.88	2.84	2.02-4.00
Unknown	77 (9.8%)	1,209 (6.8%)	-	-	-	-	-	-	-	-	-	-
**Cumulative daunorubicin dose, mg/m²**												
0	684 (87.5%)	14,630 (81.7%)	1.0	Ref.	1.0	Ref.	1.0	Ref.	1.0	Ref.	1.0	Ref.
<100	7 (0.9%)	623 (3.5%)	0.98	0.46-2.09	0.91	0.42-1.95	1.01	0.47-2.18	0.98	0.46-2.09	1.02	0.47-2.22
100-199	16 (2.0%)	953 (5.3%)	0.98	0.55-1.75	0.93	0.53-1.64	1.01	0.57-1.81	0.98	0.55-1.75	1.03	0.56-1.88
≥200	17 (2.2%)	645 (3.6%)	1.22	0.69-2.17	1.14	0.64-2.03	1.24	0.69-2.20	1.22	0.68-2.16	1.28	0.71-2.32
Unknown	58 (7.4%)	1,052 (5.9%)	-	-	-	-	-	-	-	-	-	-
**Epipodophyllotoxins**												
No	611 (78.1%)	13,434 (75.0%)			1.0	Ref.						
Yes	78 (10.0%)	3,346 (18.7%)			1.33	0.98-1.80						
Unknown	93 (11.9%)	1,123 (6.3%)			-	-						
**Vinca alkaloids**												
No	269 (34.4%)	6,194 (34.6%)					1.0	Ref.				
Yes	420 (53.7%)	10,586 (59.1%)					0.86	0.68-1.09				
Unknown	93 (11.9%)	1,123 (6.3%)					-	-				
**Platinum compounds**												
No	642 (82.1%)	14,219 (79.4%)							1.0	Ref.		
Yes	47 (6.0%)	2,561 (14.3%)							0.99	0.68-1.44		
Unknown	93 (11.9%)	1,123 (6.3%)							-	-		
**Antimetabolites**												
No	504 (64.5%)	10,376 (58.0%)									1.0	Ref.
Yes	185 (23.7%)	6,404 (35.8%)									0.92	0.72-1.18
Unknown	93 (11.9%)	1,123 (6.3%)									-	-

CI = Confidence interval; HR = Hazard ratio; No. = number; SBC = Subsequent breast cancer

[a]The base multivariable model included cumulative doxorubicin dose (categorical variable), cumulative daunorubicin dose (categorical variable), epirubicin (yes/no), age at primary cancer diagnosis (categorical variable), the combination of the chest radiation field and the associated maximum dose (categorical variable), pelvic radiation dose ≥5 Gy (yes/no), and alkylating agent cumulative exposure (cyclophosphamide equivalent dose, categorical variable). Because there is only evidence for associations between anthracyclines and alkylating agents on SBC risk, we applied the following selection procedure to evaluate other chemotherapeutic agents: we added binary indicators for epipodophyllotoxins, vinca alkaloids, platinum compounds, and antimetabolites to the base model; if addition of each variable changed any HRs of doxorubicin dose and/or daunorubicin dose by >10% compared to a model without the variable, it was included in the final models.

[b]One survivor had a SBC prior to five years after primary cancer.
