# Supplementary material for: Subsequent female breast cancer risk associated with anthracycline chemotherapy for childhood cancer
Source: Nat Med. 2023 Sep 11;29(9):2268–77. doi: 10.1038/s41591-023-02514-1 (PMC10504074; doi:10.1038/s41591-023-02514-1)
Supplement: Supplementary file 2 — Reporting Summary [file 41591_2023_2514_MOESM2_ESM.pdf]

nature portfolio

Corresponding author(s): Yuehan Wang

Last updated by author(s): Jul 20, 2023

# Reporting Summary

Nature Portfolio wishes to improve the reproducibility of the work that we publish. This form provides structure for consistency and transparency in reporting. For further information on Nature Portfolio policies, see our Editorial Policies and the Editorial Policy Checklist.

## Statistics

For all statistical analyses, confirm that the following items are present in the figure legend, table legend, main text, or Methods section.

| n/a | Confirmed | |
|---|---|---|
| ☐ | ☒ | The exact sample size (*n*) for each experimental group/condition, given as a discrete number and unit of measurement |
| ☒ | ☐ | A statement on whether measurements were taken from distinct samples or whether the same sample was measured repeatedly |
| ☐ | ☒ | The statistical test(s) used AND whether they are one- or two-sided<br>*Only common tests should be described solely by name; describe more complex techniques in the Methods section.* |
| ☐ | ☒ | A description of all covariates tested |
| ☐ | ☒ | A description of any assumptions or corrections, such as tests of normality and adjustment for multiple comparisons |
| ☐ | ☒ | A full description of the statistical parameters including central tendency (e.g. means) or other basic estimates (e.g. regression coefficient) AND variation (e.g. standard deviation) or associated estimates of uncertainty (e.g. confidence intervals) |
| ☐ | ☒ | For null hypothesis testing, the test statistic (e.g. *F*, *t*, *r*) with confidence intervals, effect sizes, degrees of freedom and *P* value noted<br>*Give P values as exact values whenever suitable.* |
| ☒ | ☐ | For Bayesian analysis, information on the choice of priors and Markov chain Monte Carlo settings |
| ☒ | ☐ | For hierarchical and complex designs, identification of the appropriate level for tests and full reporting of outcomes |
| ☒ | ☐ | Estimates of effect sizes (e.g. Cohen's *d*, Pearson's *r*), indicating how they were calculated |

*Our web collection on statistics for biologists contains articles on many of the points above.*

## Software and code

Policy information about availability of computer code

| | |
|---|---|
| Data collection | Data formatting was performed by R software (version 4.0.3). |
| Data analysis | All analyses were conducted in R software (version 4.0.3). The statistical code for our main analyses is available at https://github.com/yuehanwanggitub/IPDanthracyclines.git. |

For manuscripts utilizing custom algorithms or software that are central to the research but not yet described in published literature, software must be made available to editors and reviewers. We strongly encourage code deposition in a community repository (e.g. GitHub). See the Nature Portfolio guidelines for submitting code & software for further information.

## Data

Policy information about availability of data

All manuscripts must include a data availability statement. This statement should provide the following information, where applicable:

- Accession codes, unique identifiers, or web links for publicly available datasets
- A description of any restrictions on data availability
- For clinical datasets or third party data, please ensure that the statement adheres to our policy

The International Consortium for Pooled Studies on Subsequent Malignancies after Childhood and Adolescent Cancer database is not an open-access database due to ethical and data protection constraints. The pseudonymized data is managed by the Princess Máxima Center for Pediatric Oncology in the Netherlands and cannot be shared with investigators outside the institute without consent from all involved parties. However, potential collaborators are welcome to submit

proposals to Dr. Jop Teepen (J.C.Teepen@prinsesmaximacentrum.nl), which will be considered by the consortium. The consortium will come up with a decision about submitted proposals within three months after application. The country-specific female breast cancer rates of the Cancer Incidence in Five Continents database were used as the general female population in our study. These data are publicly available: https://ci5.iarc.fr/.

## Human research participants

Policy information about studies involving human research participants and Sex and Gender in Research.

| | |
|---|---|
| Reporting on sex and gender | Only female childhood cancer survivors were included in this study because the study outcome was subsequent female breast cancer risk. |
| Population characteristics | Specific demographic and treatment characteristics of included female five-year childhood cancer survivors were presented in Table 1, Supplementary Table 1 and 2. Briefly, among the eligible 17,903 five-year survivors, the median age at primary childhood cancer diagnosis was 6.7 years (interquartile range (IQR) 2.8-13.0). In total, 782 survivors developed a first subsequent breast cancer at a median age of 39.7 years (IQR 34.3-44.9), including 616 invasive breast cancer (IBC) and 166 ductal carcinoma in situ (DCIS) cases. The median attained age at the end of follow-up was 33.7 years (IQR 25.9-41.6). |
| Recruitment | Data in Europe and North America with available data on radiotherapy cumulative dose and fields and cumulative dose for chemotherapy was collected, including the following six studies: five cohort studies (Childhood Cancer Survivor Study (CCSS, 9,671 women diagnosed in period 1970-1999), St. Jude Lifetime Cohort Study (SJLIFE, 2,236 women diagnosed in period 1962-2012), Dutch Childhood Cancer Survivor Study LATER (DCCSS LATER, 2,237 women diagnosed in period 1963-2001), French Childhood Cancer Survivor Study (FCCSS, 3,415 women diagnosed in period 1943-2000) and Dutch Hodgkin Late Effects cohort (DHL, 265 women diagnosed in period 1965-1995)), and one case-cohort study (Swiss Childhood Cancer Survivor Study (SCCSS, 79 women diagnosed in period 1976-2007)) . |
| Ethics oversight | This study involves human participants. The contributing cohort study teams obtained institutional review board and/or Ethics Committee approval or exemption in their respective contributing institute. The pooling effort is exempt from review in compliance with Dutch law and regulations for health research involving human beings. Data sharing agreements between the Princess Máxima Center for Pediatric Oncology and all data providers are in place. Written consent was obtained from all patients of the CCSS and the SJLIFE. Specific informed consent for retrospective data collections for selected groups of patients for the DCCSS-LATER, the Dutch Hodgkin Late Effects cohort, the FCCSS and the SCCSS cohorts was waived in accordance with the country's legislation. |

Note that full information on the approval of the study protocol must also be provided in the manuscript.

# Field-specific reporting

Please select the one below that is the best fit for your research. If you are not sure, read the appropriate sections before making your selection.

☒ Life sciences ☐ Behavioural & social sciences ☐ Ecological, evolutionary & environmental sciences

For a reference copy of the document with all sections, see nature.com/documents/nr-reporting-summary-flat.pdf

# Life sciences study design

All studies must disclose on these points even when the disclosure is negative.

| | |
|---|---|
| Sample size | Data from five cohort studies (Childhood Cancer Survivor Study (CCSS), St. Jude Lifetime Cohort Study (SJLIFE), Dutch Childhood Cancer Survivor Study LATER (DCCSS LATER), French Childhood Cancer Survivor Study (FCCSS) and Dutch Hodgkin Late Effects cohort (DHL)), and one case-cohort study (Swiss Childhood Cancer Survivor Study (SCCSS)) in Europe and North America with available data on radiotherapy cumulative dose and fields and cumulative dose for chemotherapy was pooled together. |
| Data exclusions | Female childhood cancer survivors not meeting the following criteria were excluded in the pooled study: a primary cancer diagnosis at <21 years of age, and survival ≥5 years from diagnosis. |
| Replication | N/A. This study is a pooled analysis of clinical data from various cohort studies. Replication, as in for preclinical work, is therefore not applicable here. |
| Randomization | N/A. This study is an observational study; therefore, randomization is not applicable here. Anthracycline-related breast cancer risks were adjusted for other covariates which have been shown or suggested to be associated with breast cancer risk in previous studies, such as chest radiotherapy, pelvic radiotherapy, age at primary cancer diagnosis, and alkylating agents. |
| Blinding | Our data were collected from data providers of each included study. No control/placebo arm included in our study, therefore, blinding was not applicable. |

# Reporting for specific materials, systems and methods

We require information from authors about some types of materials, experimental systems and methods used in many studies. Here, indicate whether each material, system or method listed is relevant to your study. If you are not sure if a list item applies to your research, read the appropriate section before selecting a response.

## Materials & experimental systems

| n/a | Involved in the study |
|---|---|
| ☒ | ☐ Antibodies |
| ☒ | ☐ Eukaryotic cell lines |
| ☒ | ☐ Palaeontology and archaeology |
| ☒ | ☐ Animals and other organisms |
| ☒ | ☐ Clinical data |
| ☒ | ☐ Dual use research of concern |

## Methods

| n/a | Involved in the study |
|---|---|
| ☒ | ☐ ChIP-seq |
| ☒ | ☐ Flow cytometry |
| ☒ | ☐ MRI-based neuroimaging |
